# Molecular Mechanisms and Signaling Pathways Involved in Sertoli Cell Proliferation

**DOI:** 10.3389/fendo.2019.00224

**Published:** 2019-04-16

**Authors:** Silvina Beatriz Meroni, María Noel Galardo, Gustavo Rindone, Agostina Gorga, María Fernanda Riera, Selva Beatriz Cigorraga

**Affiliations:** Centro de Investigaciones Endocrinológicas “Dr César Bergadá” CONICET-FEI-División de Endocrinología Hospital de Niños Ricardo Gutiérrez, Buenos Aires, Argentina

**Keywords:** Sertoli cells, proliferation, maturation, signal transduction, xenobiotics

## Abstract

Sertoli cells are somatic cells present in seminiferous tubules which have essential roles in regulating spermatogenesis. Considering that each Sertoli cell is able to support a limited number of germ cells, the final number of Sertoli cells reached during the proliferative period determines sperm production capacity. Only immature Sertoli cells, which have not established the blood-testis barrier, proliferate. A number of hormonal cues regulate Sertoli cell proliferation. Among them, FSH, the insulin family of growth factors, activin, and cytokines action must be highlighted. It has been demonstrated that cAMP/PKA, ERK1/2, PI3K/Akt, and mTORC1/p70SK6 pathways are the main signal transduction pathways involved in Sertoli cell proliferation. Additionally, c-Myc and hypoxia inducible factor are transcription factors which participate in the induction by FSH of various genes of relevance in cell cycle progression. Cessation of proliferation is a pre-requisite to Sertoli cell maturation accompanied by the establishment of the blood-testis barrier. With respect to this barrier, the participation of androgens, estrogens, thyroid hormones, retinoic acid and opioids has been reported. Additionally, two central enzymes that are involved in sensing cell energy status have been associated with the suppression of Sertoli cell proliferation, namely AMPK and Sirtuin 1 (SIRT1). Among the molecular mechanisms involved in the cessation of proliferation and in the maturation of Sertoli cells, it is worth mentioning the up-regulation of the cell cycle inhibitors p21Cip1, p27Kip, and p19INK4, and of the gap junction protein connexin 43. A decrease in Sertoli cell proliferation due to administration of certain therapeutic drugs and exposure to xenobiotic agents before puberty has been experimentally demonstrated. This review focuses on the hormones, locally produced factors, signal transduction pathways, and molecular mechanisms controlling Sertoli cell proliferation and maturation. The comprehension of how the final number of Sertoli cells in adulthood is established constitutes a pre-requisite to understand the underlying causes responsible for the progressive decrease in sperm production that has been observed during the last 50 years in humans.

## Introduction

Sertoli cells represent one of the most complex cells in the organism. Not only because of their three-dimensional structure but also due to their function to create a unique environment which allows germ cell development. The concept of “nurse cells” is widely used to refer to this cell type as they create a complete lining within the tubular walls which envelope spermatogenic cells. They also, by virtue of tight junction formation, constitute the main component of the blood testis barrier (BTB).

Positive correlations between total Sertoli cell number and the daily sperm production in several species, including humans, have been reported (1–3). This relationship exists because each Sertoli cell is able to sustain a limited number of germ cells (4). Thus, it may be concluded that appropriate development of the Sertoli cell population, with respect to their number and functionality, will determine spermatogenic capacity through adulthood.

Only immature Sertoli cells proliferate and, even when there are differences between species as to the pre-dominant periods of mitotic activity, it is generally accepted that proliferation stops at puberty in most species (5). Thus, the regulation of Sertoli cell proliferation—determining the final Sertoli cell number—and cessation of the proliferation concomitant with maturation—establishing adequate cell function—constitutes the foundation of adult testicular function and occurs in fetal and early postnatal life.

From the initial studies of Sertoli (6) significant advances have been made in understanding the functionality of this cell type. It is well-known that the gonadotropin Follicle-Stimulating Hormone (FSH) and also androgens regulate the proliferation and functional maturation of this cell type. In addition to these classical hormones, a great number of locally produced factors participate in the regulation of Sertoli cells reflecting one of the most representative examples of cell-cell communication (7).

Noticeably, while a huge number of reports deal with the regulation of several aspects of Sertoli cell physiology and at least two books of biblical proportions have been published (8, 9), much less is known on the regulation of proliferation of this cell type. Clermont and Perey (10) performed the earliest quantitative study of Sertoli cell proliferation based on the analysis of the percentage of Sertoli cells undergoing mitosis. More than 10 years later, Steinberger and Steinberger (11) utilizing organ culture pulsed with [^3^H]-thymidine and determining the labeling index showed that this index decreased with the age of the animal. A few years later, Griswold et al. (12) showed that FSH stimulates DNA synthesis and mitosis of immature Sertoli cells in culture and that mitotic activity is limited to immature cells. Furthermore, studies performed by Orth (13, 14) identified fetal and postnatal life in the rat as moments of high mitotic activity. From these initial studies that established the basic concepts on Sertoli cell proliferation a great number of investigators have tried to deeply understand the molecular mechanisms underlying this physiological process.

Increasing evidence for the quantitative and qualitative decline in human sperm over the past few decades has been presented (15–17). A recent study by Levine et al. (18) reported a 50–60% decline in sperm counts. The reasons for this decline are not clear yet, but modern lifestyle may be a cause (19–22). A variety of factors including those that affect Sertoli cell proliferation and maturation at cessation of mitosis might be somehow related to the impairment observed in seminiferous tubule function. Our review will highlight molecular mechanisms related to the above-mentioned processes, i.e., proliferation and maturation, which may be affected by exposure to certain therapeutic drugs or pollutants.

## Main Factors Involved in the Stimulation of Sertoli Cell Proliferation

Even though a considerable variation in the number of Sertoli cells among members of the same specie exists, it is assumed that the final number of Sertoli cells in adults results from events in fetal, neonatal or peripubertal life (23). Therefore, the hormones and locally produced factors as well as signaling pathways and molecular mechanisms involved in the stimulation of Sertoli cell proliferation are crucial to define sperm production in adult animals. Current knowledge of the roles FSH, the insulin family of growth factors, the activins and cytokines play in Sertoli cell proliferation will be summarized in the following sections.

### Follicle-Stimulating Hormone

FSH is a gonadotropin synthesized and secreted by the gonadotropic cells of the anterior pituitary gland. FSH consists of two different glycoprotein subunits: a common α subunit, which is present in other pituitary hormones, and a specific β subunit, which confers its specific biologic action (24). FSH is a central regulator of reproductive function in mammals. Physiological effects of FSH are mediated by its association with the FSH receptor (FSHR), a seven-transmembrane-domain protein, which belongs to the G protein-coupled receptor (GPCR) superfamily (25, 26). It is widely accepted that FSHR in the male is expressed exclusively in Sertoli cells (27). Consistent with the presence of receptors, FSH actions can be demonstrated during fetal life and throughout postnatal lifespan. However, the physiological response to FSH varies depending on the state of maturation of Sertoli cells (5). FSH regulates Sertoli cell proliferation only during fetal and early postnatal life, whereas it regulates differentiation after cessation of mitosis at puberty. The first demonstration of a stimulatory role of FSH in the proliferation of Sertoli cells came from studies of Griswold et al. (12, 28, 29). Other pioneering studies that established the relevance of FSH in the regulation of Sertoli cell proliferation consisted of *in vivo* procedures that lead to diminished endogenous FSH levels -decapitation *in utero* or addition of FSH antiserum to rat fetuses. These experiments showed that, as a result of lower FSH levels, incorporation of [^3^H]-thymidine in Sertoli cells decreased (14). In these studies, it was also shown that FSH increases the number of Sertoli cells in organ culture. In addition, it was shown that hemicastration of 3-day-old rats evokes enhanced Sertoli cell proliferation in the remaining testis that is accompanied by elevated levels of FSH, and that testosterone administration abrogates the compensatory hypertrophy (30). This negative effect of testosterone on Sertoli cell proliferation was interpreted to be a consequence of the negative feedback on FSH secretion that testosterone exerts. The importance of FSH in the regulation of Sertoli cell proliferation was further confirmed by a study conducted by Almirón and Chemes (31). The latter authors observed that Sertoli cell mitotic index was reduced in immature rats with FSH withdrawal accomplished by administration of high doses of testosterone propionate, and that the index increased when FSH levels were restored by injection of human FSH. Years later, the results obtained utilizing gonadotropin-deficient hypogonadal (hpg) mice treated with recombinant FSH (32, 33) or hpg mice expressing transgenic FSH (34, 35) strengthened the role of FSH in the regulation of Sertoli cell proliferation. Complementarily, a reduction in Sertoli cell number in mice with a null mutation in *Fshr* gene was observed (36–38).

Once the mitogenic role of FSH was convincingly demonstrated, further studies focused on elucidating signal transduction pathways involved in the regulation of Sertoli cell proliferation triggered by the hormone. For more than 20 years, it had been widely accepted that the canonical Gs/cyclic adenosine monophosphate (cAMP)/cAMP-dependent kinase (PKA) pathway was the unique mechanism that contributed to FSH actions (39, 40). The increase in [^3^H]-thymidine incorporation in immature Sertoli cells caused by dibutyryl-cAMP (dbcAMP) incubations (14, 29) was the first evidence for the participation of cAMP-dependent pathways in the regulation of Sertoli cell proliferation. Nowadays, growing evidence indicates the complexity associated with FSH-induced cellular signaling (41, 42). Crépieux et al. (43) showed that FSH activates the extracellular signal-regulated protein kinases 1 and 2 (ERK1/2) pathway following dual coupling of the FSHR both to Gs and to Gi heterotrimeric proteins, in a PKA- and also Src-dependent manner, leading to cell cycle progression through cyclin D1 induction and the concomitant proliferation of Sertoli cells from immature rats. The complexity of the signaling network triggered by FSHR is also reflected by the activation of phosphatidyl-inositide-3 kinase (PI3K)/Akt/p70 S6 kinase (p70S6K) by FSH in proliferating Sertoli cells (44). More recently, Riera et al. (45) showed that FSH regulates proliferation through PI3K/Akt/mammalian target of rapamycin complex 1 (mTORC1) signaling pathway. At the molecular level, an increase in phosphorylated (P)-Akt, P-mTOR, and P-p70S6K levels induced by FSH in proliferative Sertoli cells was observed. Additionally, FSH increased the levels of P-PRAS40, a substrate of Akt and a component of the mTORC1, probably contributing to improving mTORC1 signaling. Furthermore, the decrease in FSH-stimulated P-Akt, P-mTOR, P-p70S6K, and P-PRAS40 levels in the presence of a PI3K specific inhibitor emphasized the participation of PI3K in FSH signaling. Additionally, the inhibition of FSH-stimulated Sertoli cell proliferation by the effect of specific inhibitors of PI3K and mTOR confirmed the relevance of the PI3K/Akt/mTORC1 signaling pathway in the mitotic activity of FSH. These authors also showed that FSH decreased the levels of P-AMP-activated protein kinase (AMPK)—serine/threonine protein kinase that antagonizes mTORC1 actions—and concluded that AMPK-dependent mechanisms counteract FSH proliferative effects.

The intricate signaling actions of FSH are responsible for extensive alterations in the expression of genes due to the activation of a number of transcription factors in Sertoli cells. In addition to cAMP response element binding protein (CREB) (46), FSH modulates the transcriptional activity of: NFκB (47), AP1 (48), c-Myc (49), hypoxia-inducible factor (HIF)1 (50), and HIF2 (50, 51). The majority of the latter studies on transcription, with the exception of those performed on c-Myc and HIF2, were carried out in non-proliferative Sertoli cells and did not address whether these FSH-activated transcription factors might be involved in the regulation of Sertoli cell proliferation. Concerning c-Myc, it was observed that c-Myc mRNA is clearly detectable in Sertoli cells from 8-day-old rats but hardly detectable in cells from those aged 14 and 28 days. Besides, it was observed that FSH, in a cAMP-dependent manner, stimulates c-Myc mRNA expression in Sertoli cells derived from 8- to 14-day-old rats but has almost no effect in those derived from 28-day-old rats (49). This age-dependent regulation of expression strongly suggests a role of c-Myc in immature Sertoli cells. A cAMP-dependent pathway is not the only one regulating c-Myc expression as the involvement of PI3K signaling pathway has also been demonstrated (45). As for HIFs, it was shown that FSH upregulates the expression of HIF1α and HIF2α in Sertoli cells obtained from 20-day-old rats (50), a developmental stage in which rat Sertoli cells barely proliferate and have established the BTB. On the other hand, in Sertoli cells obtained from 8-day-old rats, a stage in which Sertoli cells are actively proliferating, FSH upregulates only HIF2α levels (51). In addition, it is worth mentioning that it has been observed that HIF1 and HIF2 have the opposite effects on regulation of proliferation in other cell types—while HIF2 promotes cell cycle progression, HIF1 inhibits it (52, 53). The participation of HIF2 in the regulation of proliferation by FSH was further confirmed by the demonstration that inhibitors of HIFs down-regulate bromo-deoxyuridine (BrdU) incorporation, cyclin D1 expression and c-Myc activity stimulated by FSH in immature Sertoli cells (51).

In summary, FSH positively regulates the proliferation of Sertoli cells by activating cAMP/PKA/ERK1/2 and PI3K/Akt/mTORC1 dependent-pathways, and by increasing the transcriptional activity of c-Myc and HIF2 and the expression of cyclin D1. The characterization of some of the signaling pathways regulated by FSH has been an important step toward the understanding of how this hormone promotes, on the one hand, Sertoli cell proliferation during fetal and early postnatal life and, on the other hand, maturation after cessation of mitosis at puberty. Nevertheless, many of the mechanisms by which FSH exerts its biological actions which, as mentioned above, vary with the developmental status of the animal, remain to be fully understood. Finally, yet importantly, the involvement of FSH-promoted autocrine factors in Sertoli cell proliferation should be considered.

### Insulin Family of Growth Factors

The insulin family of growth factors—insulin, insulin like growth factors I (IGF-1) and II (IGF-2) and relaxin– are small polypeptides that are responsible for the control of growth, metabolism, and reproductive functions. IGF-1 and IGF-2, which share 70% of their amino acid sequence, are ubiquitously expressed unlike insulin that is expressed in the pancreatic islets of Langerhans β-cells. The physiological effects of these peptides are mediated through the activation of two related tyrosine kinase receptors: the insulin receptor (InsR), and the IGF-1 receptor (IGF-1R). Ligand binding activates receptor tyrosine kinase activity and subsequently, phosphorylation of tyrosine residues in the receptor itself and a set of proteins known as insulin receptor substrates (IRS) occurs. Four IRS proteins that initiate intracellular pathways, named IRS1-4, have been identified (54). Activation of the receptor will lead to stimulation of two major signaling pathways: PI3K and ERK1/2, both of which are associated with proliferation, differentiation, metabolism, and cell survival.

The members of this family that have been more extensively studied in the context of testicular function are insulin and IGF-1. Utilizing different approaches, crucial roles for both peptides in testicular development were established. In their analysis of *Igf-1* knock-out (KO) mice, Baker et al. (55) showed the importance of IGF-1 for the development and fate of the mouse male gonad. Sertoli cell characteristics were not evaluated in these initial studies. Other studies, related specifically to Sertoli cell proliferation, indicated that IGF-1 effects take place both during embryonic and neonatal periods. It has been shown that Sertoli cells isolated from embryonic mouse testis express IGF-1 and IGF-1R and that IGF-1 treatment increases BrdU incorporation and promotes cell cycle progression (56). Furthermore, IGF-1 receptors were identified in neonatal Sertoli cells (57, 58) and a positive role of IGF-1 in regulating Sertoli cell proliferation in cultured cells from different species (rat, pig, and bull) was demonstrated (57, 59–61). Moreover, cultured Sertoli cells with inactivated IGF-1R showed decreased BrdU incorporation and cyclin D2 expression and increased p21Cip1 and p53 protein levels (62). As for the role of insulin, early studies showed that insulin exerts proliferative effects as well as IGF-1, however, as higher concentrations of insulin (micromolar) than those of IGF-1 (nanomolar) were required to elicit biological responses, it was suggested that insulin acted via IGF-1 receptors (63). More recently, additional support to the concept that both peptides, IGF-1 and insulin, are involved in proliferation of Sertoli cells was provided by analyzing mice lacking *Insr* and/or *Igf1r* specifically in Sertoli cells. Adult testes of mice lacking both *Insr* and *Igf1r* in Sertoli cells (SC-*Insr*;*Igf1r*) displayed a 72% reduction in testis size and a 79% reduction in daily sperm production. Reduced proliferation of immature Sertoli cells during late fetal and early neonatal development in these animals was also observed. However, despite the marked reduction in sperm production they were fertile indicating that the absence of IGF signaling in Sertoli cells does not impair spermatogenesis (64). As a whole, these investigations established that insulin and IGF-1 are essential components of the endocrine and paracrine network that regulates Sertoli cell proliferation.

Regarding the signal transduction pathways elicited by insulin/IGF-1 system in Sertoli cells, there is general agreement that they can activate PI3K/Akt and ERK1/2 signaling pathways and, additionally, that this is mediated by IRS2. With regard to the latter, it has been shown that adult *Irs2* KO mice show a 45% reduction in testis weight, with a reduction in the number of Sertoli cells, spermatogonia, spermatocytes, elongated spermatids, and spermatozoa, whereas testicular development in *Irs1* KO mice does not evince these reductions (65). A reduced testicular size was observed as early as the neonatal period, suggesting that testicular development impairment in *Irs2* KO mice also occurs in the fetal period. Altogether, these data indicate that IRS2, by mediating IGF-1 signaling during embryonic and early postnatal periods, plays a critical role in testicular development. The role of the ERK1/2 pathway in mediating IGF-1 effects on Sertoli cell proliferation had been suggested in experiments showing that the effects of IGF-1 on embryonic Sertoli cell proliferation were inhibited by a specific ERK1/2 pathway inhibitor (56). Regarding PI3K/Akt pathway, it has been shown that IGF-1 can stimulate this pathway in immature Sertoli cells (60). However, a direct relationship of the activation of PI3K/Akt pathway in mediating IGF-1 action in Sertoli cell proliferation has not been determined yet.

Interestingly, the insulin/IGF-1 signaling pathway has been proposed to play a role in mediating FSH effects in immature Sertoli cells. Initial studies on the regulation of IGF-1 system by FSH demonstrated that this gonadotropin stimulates IGF-1 and inhibits IGF binding protein (IGFBP) 3 secretion in Sertoli cells (66–69). By the fact that, in *Fshr* KO mice, testis weight, and Sertoli cell numbers were reduced ~50% (70), while in SC-*Insr;Igf1r* KO mice, a greater reduction was observed (64), it has been suggested that the effects of FSH on the proliferation and differentiation of immature Sertoli cells are mediated, at least in part, by IGF-1. Pitetti et al. (64) studied the potential interaction between FSH and the insulin/IGF signaling pathway. The authors utilized an experimental model consisting of neonatal hemicastration of wild type and SC-*Insr;Igf1r* KO animals. While testis size and epididymal sperm counts were increased in hemicastrated wild type males, no effects were observed in hemicastrated SC-*Insr;Igf1r* mutant mice. Moreover, human recombinant FSH therapy did not increase testicular size and sperm output in SC-*Insr;Igf1r* KO mice. Based on these results the authors suggested that FSH requires the insulin/IGF-1 signaling pathway to mediate its proliferative effect on immature Sertoli cells. However, it has to be kept in mind that in these SC-*Insr;Igf1r* KO mice a reduction in FSHR and Akt signaling was also observed. Thus, the inability of Sertoli cells to proliferate after hemicastration or after FSH treatment might be accounted for by the reduction in both FSHR and Akt signaling. In summary, it can be concluded that the effects of FSH on immature Sertoli cells can be mediated, at least in part, by IGF-1 and that local production of IGF-1 is an important component of the intratesticular network involved in the regulation of Sertoli cell number, testis size and sperm output in mammals.

Relaxin is another member of the insulin-related peptide family involved in Sertoli cell proliferation. This peptide was first recognized for its important role during pregnancy and parturition (71, 72). Relaxin is structurally similar to insulin, but binds to GPCRs termed the relaxin family peptide receptors (RXFP 1 and 2) (73). Relaxin mRNA levels are higher in the testis of immature rats than in the adult ones, which suggests an important role in an early period of life (74). Considering that relaxin and RXFP1 expression is found in immature Sertoli cells, an autocrine regulation has been predicted (75). Relaxin increases the incorporation of [^3^H]-thymidine and the levels of proliferating cell nuclear antigen (PCNA) in Sertoli cell cultures. Relaxin-induced Sertoli cell proliferation involves activation of a Gi protein and activation of EKR1/2 and PI3K/Akt pathways (76). Supporting the hypothesis that relaxin has a role on Sertoli cell proliferation, it has been observed that KO mice for this gene have smaller testes (77). More recently, a crosstalk between FSH and relaxin at the end of the proliferative stage in rat Sertoli cells was demonstrated, and the authors postulate that whereas FSH action predominates and seems essential to direct cell maturation, relaxin seems to preferentially promote Sertoli cell proliferation (78, 79).

### Activins and Inhibins

The gonadal peptides activins and inhibins belong to the transforming growth factor (TGF) β superfamily, and have important roles in reproduction and development. Activins and inhibins were discovered and named based on their abilities to stimulate or inhibit FSH release by gonadotrophs (80, 81). Activins are homodimers of two β inhibin subunits encoded by five genes designated *Inhba* to *Inhbe* (82–85). The most studied activins are those called activin A (βAβA) and activin B (βBβB). On the other hand, inhibins are heterodimers of one of the β subunits –βA or βB– with a common inhibin α-subunit, encoded by *Inha* gene, namely inhibin A (αβA), and inhibin B (αβB), respectively (82, 86).

All information related to activin regulation of Sertoli cell function has been obtained using activin A. Activin A is expressed in fetal and postnatal testis with variable cell localizations (87–91). In the fetal testis, Leydig cells are the main source of activin A in the mouse and human (92, 93). Barakat et al. (94) showed that Inh βA subunit is detected by immunohistochemistry in Sertoli cells, Leydig cells, peritubular myoid cells (PTMC), and different types of germ cells. This latter study, which included studies in newborn mice, also showed that testicular concentration of activin A is high during the period of Sertoli cell proliferation and then decreases to reach a low value that remains constant until adulthood. Considering that Buzzard et al. (89) had previously shown that neonatal PTMC in culture produce higher levels of activin A than Sertoli cells, it has been proposed that the high concentrations of activin A found in neonatal testis are due to activin A produced by PTMC.

Activin A signaling is mediated by binding to a type II receptor subunit, either ActRIIa or ActRIIb, which causes type II receptor to phosphorylate type I receptor, ActRIb (ALK4). Phosphorylated type I receptor recruits and phosphorylates SMAD2 and/or SMAD3, also called regulatory SMADs. The latter phosphorylated proteins dissociate from the receptor and oligomerize with the common SMAD4. This oligomer translocates to the nucleus and affects specific gene transcription. The relevance that activin A may have in Sertoli cell mitosis is pointed out by the presence of type II and type I receptors in mitotically active Sertoli cells (89, 95).

More than 20 years ago, at the time when the TGFβ peptide superfamily was being characterized, the need to investigate possible paracrine effects within the testis became apparent. To this respect, a synergistic effect of activin A with FSH on Sertoli cell proliferation in the neonatal period was described (89, 96). The effect of activin A on fetal Sertoli cell mitosis was analyzed in *Inhba* KO mice. *Inhba* KO males have significantly fewer Sertoli cells and a lower testis weight than wild type males at birth. Concomitantly, a reduction in BrdU- and PCNA-positive Sertoli cells and a decrease in cyclin D2 expression in Sertoli cells in these animals were observed (97). A study using genetic disruption of *Inhba* specifically in fetal Leydig cells (*Inhba* cKO) showed decreased proliferation of Sertoli cells, and the authors postulated that activin A produced by Leydig cells is the relevant paracrine regulator of fetal Sertoli cell mitosis (93). Noticeably, it has been shown that activin A is present in fetal human testis and that activin A increases Sertoli cell proliferation in fetal human testis in culture (92, 98). These results point out the physiological relevance of this peptide in fetal Sertoli cell mitosis.

The role of different activin receptors and of the SMAD signaling pathway in the proliferative effect of activin A has also been studied. It has been shown that mice with deletion of *ActrIIa* have smaller testis size and a reduced number of Sertoli cells (99). On the other hand, abrogation of ALK4/5/7 signaling in gonad cultures has been shown to promote a significant reduction in fetal Sertoli cell proliferation (100). As for the participation of SMAD signaling pathway, Itman et al. (101) showed that activin A promotes nuclear accumulation of SMAD3 rather than SMAD2 in proliferative Sertoli cell cultures. These results suggest that activin A signals preferentially through SMAD3 in immature Sertoli cells. Moreover, mice with conditional deletion of *Smad4* in Sertoli cells showed decreased Sertoli cell proliferation and a reduction in testis size (93). Altogether, these studies support the notion that activin receptors and SMAD3-SMAD4 signaling dependent pathways are involved in the regulation of Sertoli cell proliferation.

As mentioned before, activin A production decreases with the age of the animal. The most marked drop occurs at puberty at the time that Sertoli cells mature and are in a non-proliferative state (94). Even though activin A production decreases, it is present throughout adulthood, and investigators searched for a role of the peptide in maturing Sertoli cells. In this context, Nicholls et al. (102) using Sertoli cell cultures that have hallmarks of mature cells—tight junction formation, mitotic arrest and expression of maturity markers—showed that activin A inhibits tight junction formation between neighboring Sertoli cells. In addition, the authors showed that activin A induces proliferation of these mature Sertoli cells and increases cytokeratin 18 expression, a marker of immature Sertoli cells. These *in vitro* studies were complemented with an *in vivo* approach. Adult mice with increased systemic activin A levels showed a disruption of the BTB, an increase in the number of seminiferous tubules with severe spermatogenic defects and a decrease in testis weight. The authors concluded that the switch from high to low activin A levels during testis development is physiologically relevant and might be important for appropriate Sertoli cell function and fertility.

The role of activin B on Sertoli cell function is much less understood. Studies using *Inhbb* KO mice suggest that activin B is not involved in Sertoli cell proliferation or testicular development (103). However, further studies will be necessary to determine if activin B has any role in the regulation of Sertoli cell proliferation.

Regarding the inhibins, it has been shown that inhibin B is the major circulating inhibin in males and that it is produced mainly by Sertoli cells in the testis (92, 94, 104). Mice with a deletion of *Inha* have normal testicular development during embryogenesis but develop testicular tumors by 30 days of age. These tumors completely alter testicular architecture and render the mice infertile (105). Based on the latter observations, a marked inhibition of Sertoli cell proliferation by inhibin B was initially postulated. A few years later, it was shown that *Inha* KO mice, which do not produce inhibin B, have overproduction, and unbalanced action of activin A that might be responsible for the observed phenotype (106). In support of the latter hypothesis, it has been observed that the genetic deletion of *Smad3*, an important activin A signaling molecule, relieves the Sertoli cell tumor-forming phenotype of *Inha* KO mice (107, 108). Altogether, this evidence suggests that in adulthood inhibin B has no role by itself but plays a role in the modulation of activin A-induced Sertoli cell proliferation.

In summary, activin A stimulates Sertoli cell proliferation during fetal and postnatal period. Increasing levels of inhibin B at puberty may counteract activin A effects.

### Cytokines

Cytokines are typically characterized as factors made by more than one cell type that act locally in an autocrine or paracrine fashion and play a pivotal role in the regulation of immune and non-immune cells. Different cell types in the testis under both physiological and pathological conditions produce cytokines (109, 110). Considering that the mammalian testis is a notable immune-privileged site, which protects haploid immunogenic germ cells from the harmful effects of immune responses, most studies focused on the effects of cytokines on the maintenance or disruption of the immune environment. However, a few addressed cytokine regulation of Sertoli cell proliferation.

Interleukin 1 (IL-1) exists as two major agonist isotypes, IL-1α, and IL-1β, and these cytokines have a naturally antagonist named IL-1 receptor antagonist (IL-1ra). In rodent testis, IL-1α, and IL-1β are produced by Sertoli cells, interstitial macrophages and Leydig cells (111–115). The production of IL-1α by human Sertoli and Leydig cells has also been demonstrated (116). Both IL-1s exert their effects by binding IL-1 receptor type I that is constitutively expressed in Sertoli cells (117, 118). As for Sertoli cell proliferation, it has been shown that IL-1α and IL-1β increase DNA synthesis and Sertoli cell number *in vitro* and that IL-1α has a more potent effect than IL-1β (118). Petersen et al. (119) explored the signaling pathways activated by IL-1α and their participation in the proliferative effects on Sertoli cells. The authors showed that IL-1α activates p38 MAPK and JNK pathways but not the ERK1/2 cascade in immature Sertoli cells, and that the p38 MAPK pathway mediates the mitogenic effect of IL-1α. IL-1α expression can be demonstrated at about 20 days of age increasing thereafter in the rat testis (120). Tumor necrosis factor (TNF) α is another cytokine with known effects in the pro-inflammatory and immunoregulatory responses, and apoptosis. TNFα is produced by interstitial macrophages, spermatocytes and spermatids in the adult testis (121). Only TNF receptor 1 has been detected in Sertoli cells and probably mediates TNFα biological actions (122). Petersen et al. (123) presented data consistent with a possible positive role of TNFα on Sertoli cell proliferation. Considering that neither IL-1α nor TNFα were detected in the immature testis, the possible role of these cytokines in Sertoli cell proliferation under physiological conditions is at least arguable.

In summary, the effects of cytokines on Sertoli cell proliferation under physiological conditions are unlikely, however, they may have some relevance under pathological conditions with elevated intratesticular cytokine levels.

## Main Factors Involved in Cessation of Proliferation and in Maturation of Sertoli Cell

Cessation of proliferation of Sertoli cells is accompanied by a maturation process that consists in profound changes in gene expression, BTB establishment and the acquisition of full capacity to sustain developing germ cells. Therefore, the analysis of hormones and locally produced factors as well as the analysis of the signaling pathways and molecular mechanisms involved in cell cycle arrest and maturation of Sertoli cells are also relevant to the understanding of possible alterations in sperm production. Current knowledge of the role of androgens, estrogens, thyroid hormones, retinoic acid, and opioids in the cessation of proliferation and/or maturation will be summarized in the following sections.

### Androgens

The role of androgens in male fertility and in the maintenance of spermatogenesis is well-known and has been extensively reviewed (124, 125). During embryogenesis, fetal Leydig cells secrete testosterone shortly after differentiation to ensure virilization of the male embryo, and this secretion gradually declines preceding birth (126). Another testosterone surge occurs following birth and the profile of this neonatal testosterone surge has been characterized in different species (127). Then, testosterone decreases to very low levels until the onset of puberty (128, 129). At puberty, serum testosterone rises again reaching adult levels that cause development of secondary sex characteristics and progressive acquisition of reproductive capacity.

Androgens exert most of their effects through genomic actions, which involve diffusion through the plasma membrane to bind the androgen receptor (AR), which is sequestered by heat shock proteins in the cytoplasm. The interaction of the steroid with the AR leads to a conformational change in AR which causes its release from heat shock proteins. Ligand-bound AR then translocates to the nucleus where it interacts with androgen response elements (AREs) in gene promoter regions, recruiting co-regulator proteins and regulating gene transcription. An extensive review dealing with the mechanism by which AR acts as a ligand-dependent transcription factor was published by Heemers and Tindall (130).

AR is widely expressed in the rat testis, specifically in Sertoli, Leydig, and PTMC (131). The localization of AR in germ cells is controversial, with some studies showing absence of AR (131–134) and other studies presenting evidence of AR expression in germ cells (135–137). AR is absent in fetal rat Sertoli cells, and its expression becomes progressively stronger during postnatal development (131, 138, 139).

It was generally believed that androgens played little if any role in Sertoli cell proliferation in rodents, primarily because the AR is weakly expressed in Sertoli cells during early postnatal life. Precisely, the fact that the treatment of neonatal mice with testosterone propionate did not modify the expression of proliferation markers—c-Myc and PCNA—in immature Sertoli cells was interpreted as a consequence of the low levels of expression of AR (140). This hypothesis was questioned by studies in testicular feminized mice (Tfm, nonsense mutation in *Ar* gene resulting in a complete absence of nuclear receptor protein in all tissues) showing a decrease in the number of Sertoli cells in adulthood (141). However, a controversy arose on these observations as these animals suffer cryptorchidism that may be the cause of reduced Sertoli cell number. A year later, Atanassova et al. (142) also postulated a positive effect of androgens on Sertoli cell proliferation. These authors treated neonatal rats with the antiandrogen flutamide and observed reduced Sertoli cell number despite the presence of elevated FSH levels. The development of a Sertoli cell-selective *Ar* knockout mouse (SCARKO) opened up new possibilities for elucidating the role that androgens play in regulating Sertoli cell proliferation. In sharp contrast to what was observed in Tfm, flutamide-treated animals and ARKO—*Ar* KO mice with an analog phenotype to Tfm—models, the final number of Sertoli cells in SCARKO mice was unaltered (143). SCARKO animals conclusively evinced that AR expression in Sertoli cells is not required for attainment of a normal Sertoli cell number. Tan et al. (143) suggested that AR expression in other testicular cell types might be important for the modulatory role of testosterone on Sertoli cell proliferation. As PTMC express AR intensely throughout fetal and postnatal life, and, there is abundant evidence that PTMC secretions can modify Sertoli cell function (144, 145), it was hypothesized that androgens regulated Sertoli cell proliferation indirectly through their effects on PTMC. Interestingly, transgenic PTMC-*Ar*^−/y^ mice exhibit decreased testicular weight and sperm count, and even though the authors did not determine the number of Sertoli cells, it is reasonable to think that the reduction in germ cell number in this model might be a consequence of a diminution in the number of Sertoli cells (146). Taking into account that there is evidence that activin A produced by PTMC can stimulate Sertoli cell proliferation (89), it was proposed that activin A might be the paracrine factor involved in the indirect action of androgens. The development of a transgenic model that prematurely expresses the AR specifically in Sertoli cells (TgSCAR) enabled to demonstrate that androgens, by acting specifically on Sertoli cell AR, induce cell maturation. Sertoli cell maturation in TgSCAR mice was demonstrated by the observation of accelerated postnatal formation of seminiferous tubular lumen and for elevated levels of mRNAs coding for tight junction or phagocytic function proteins. Additionally, a premature AR expression in Sertoli cells led to a reduction of the pool of immature cells available for FSH induced mitotic expansion and resulted in fewer Sertoli cells in adulthood (147). In agreement with these findings, Buzzard et al. (148) had previously shown that testosterone inhibited rat Sertoli cell proliferation in primary cultures through the induction of cell cycle inhibitors p21Cip1 and p27Kip1.

In addition to the classical AR-mediated androgen responses, the regulation by androgens of Sertoli cell function may involve non-classical responses (149, 150). Recently, a novel membrane receptor for androgens, which is unrelated to AR, was identified. The zinc transporter ZRT-and Irt-like Protein (ZIP) 9, one of the 14 members of the solute carrier family 39 (SLC39) that regulates zinc homeostasis, is now known to mediate some androgen actions (151). To this respect, the expression of ZIP9 and its participation in the regulation of claudin-1 and—5 expression and the assembly of tight junctions have been described in a Sertoli cell line (152). Thus, the contribution of ZIP9 to androgen regulation of Sertoli cell maturation should be considered.

In summary, while androgens were initially thought to have a positive effect on Sertoli cell proliferation, studies on transgenic mice suggest that androgen-dependent regulation of Sertoli cell proliferation is an indirect effect probably exerted through the secretion of a paracrine factor. Direct effects of androgens on Sertoli cells seem to be related to maturation of this cell type. The mechanisms participating in androgen regulation of Sertoli cell function remain to be fully elucidated.

### Thyroid Hormones

Thyroid hormones T3 and T4 (TH) are critical regulators of growth, development, and metabolism in virtually all tissues. TH initiate biological responses via classical genomic pathways by binding to TH receptors (TRs) that are codified by two genes, *Tra*, and *Trb*. Alternative splicing of the RNA transcript of both genes generates several different protein isoforms. Only four of these TR isoforms, TRα1, TRβ1, TRβ2, and TRβ3, seem to bind TH and act as TRs (153, 154). Additionally, a 43-kDa truncated form of the nuclear receptor TRα1 (p43) synthesized by the use of an internal initiation site of translation in the TRα1 transcript was identified in the mitochondrial matrix (155). The p43 stimulates mitochondrial transcription and protein synthesis in the presence of T3 (156). Nongenomic effects of TH initiated by activation of a plasma membrane receptor integrin αvβ3 have also been described (157).

Testicular TR expression varies with the age of the animal, particularly in Sertoli cells. TRα1 is expressed in proliferating Sertoli cell nuclei, and its expression decreases coincident with the cessation of proliferation. On the other hand, TRβ1 mRNAs is expressed at low levels throughout lifespan and the corresponding protein is not detected (158). High-affinity binding sites for T3 have been observed in rat Sertoli cell mitochondria (159) and these results suggest the presence of the p43 receptor in this cell type. TH interactions with the integrin αvβ3 receptor, which mediate rapid responses, have also been described in Sertoli cells (160).

Concerning the role of TH in immature Sertoli cells, an extensive body of data obtained from *in vivo* and *in vitro* models shows that TH inhibit Sertoli cell proliferation and stimulate their functional maturation in prepubertal testis through its interaction with different TH receptors. The central role of TH in regulating immature Sertoli cell function was highlighted by studying the effects of hypothyroidism and hyperthyroidism on neonatal rats. On the one hand, it was shown that early hypothyroidism causes an increase in testis size and in daily sperm production in adult rats (161), which is correlated with an increment in Sertoli cell number and with a delay in Sertoli cell maturation (162). On the other hand, high neonatal TH levels reduced the period of Sertoli cell proliferation, accelerated tubular lumen formation, and increased inhibin secretion (163). These results indicated that, additionally to its role in halting proliferation, TH also promote Sertoli cell maturation. A more recent study made similar observations on Sertoli cell proliferation and maturation as a consequence of neonatal hypothyroidism and hyperthyroidism, which were also induced by propylthiouracil (PTU) and T3 treatment, respectively. The total number of Sertoli cells per testis was significantly increased in PTU-treated mice in comparison to the controls, whereas the opposite occurred in T3-treated mice. Although the meiotic index and Sertoli cell spermatogenic efficiency were similar in all three experimental groups, the total daily sperm production per testis was significantly higher and lower than in control animals in PTU- and T3-treated mice, respectively (164).

Culture of immature Sertoli cells constitutes another experimental approach that was used to analyze the role of TH in Sertoli cell proliferation and maturation. In these *in vitro* studies, it was observed that TH inhibited FSH-stimulated Sertoli cell mitosis (165, 166) and that TH inhibition of Sertoli cell proliferation was accompanied by an increase in the expression of the cell cycle inhibitors p21Cip1 and p27Kip1 (148). It is worth mentioning that studies in KO animals for these cyclin-dependent kinase inhibitors (CDKIs) demonstrated that both are important inhibitors of Sertoli cell proliferation, and that loss of these CDKIs leads to a large increase in the adult Sertoli cell population, as well as an increase in daily sperm production and in testis weight (167). On the other hand, TH treatment stimulated the expression of Sertoli cell maturation markers such as inhibin B, clusterin, and AR and inhibited the expression of immature Sertoli cell markers such as aromatase and Anti-Müllerian hormone (AMH) (165, 168–171). Taking together, the evidence indicates that TH are central players in the transition from an immature to a functionally mature Sertoli cell phenotype.

Studies in KO animals for different THR (*Tr*α KO and *Tr*β KO mice) were used to determine the roles of these receptors in mediating TH effects on Sertoli cells and testicular development (172). These studies on transgenic mice showed that TRα1, but not TRβ, is the receptor by which TH promote Sertoli cell maturation. This conclusion was further confirmed by analyzing a specific Sertoli cell *Tr*α*1* mutant animals, *Tr*α^AMI^-SC (173). Additionally, it was shown that *p43* KO mice depict a testicular phenotype very similar to that observed in *Tr*α KO and *Tr*α^AMI^-SC mice, suggesting that mitochondrial p43 receptor has a physiological role in Sertoli cell development (174).

Regarding the molecular mechanisms involved in the cessation of Sertoli cell proliferation by TH, the role of connexin 43 (Cx43), a constitutive protein of gap junctions that participates in the control of cell proliferation and tight junction formation, deserves special attention. To this respect, it was observed that the inhibitory effect of TH on Sertoli cell mitosis is associated with a time- and dose-dependent increase in Cx43 levels (175). The authors also showed that TH increased Cx43 levels in Sertoli cell cultures. Interestingly, specific Sertoli cell *Cx43* KO mice (SC-*Cx43*KO) showed sustained proliferation and delayed maturation of Sertoli cells in adulthood (176). Altogether, the results support the idea that Cx43, by promoting the tight junction formation between Sertoli cells, plays a pivotal role in the ability of TH to promote Sertoli cell maturation. The participation of p21Cip1 and p27Kip1 also merits consideration. Early studies showed that p27Kip1 levels in Sertoli cells are inversely related to the proliferative activity of these cells (177). Additionally, it has been shown that TH status affects p27Kip1 expression in neonatal Sertoli cells *in vivo* (178). It is well-known that p27Kip1 and to a lesser extent p21Cip1 are primarily regulated through changes in proteolytic degradation (179, 180), however, conclusive evidence for the relevance of this mechanism in Sertoli cell physiology has not been obtained yet. Other molecular mechanisms underlying the ability of TH to promote Sertoli cell cycle arrest are those that involve transcription factors c-Myc and JunD, activator and repressor, respectively of cyclin-dependent kinase 4 (CDK4) expression. By utilizing *Tr*α^AMI^-SC and *p43* KO animals, up-regulation of CDK4 and c-Myc was observed and it was postulated that these proteins are main factors controlling the proliferation of Sertoli cells (173, 174). However, direct effects of TH on c-Myc expression or on transcriptional activity in Sertoli cells remain to be determined.

The participation of signal transduction pathways in TH effects on immature Sertoli cells was scarcely analyzed. To this respect, Sun et al. (181) postulated that the effects of TH on Sertoli cell proliferation are dependent on inhibition of PI3K/Akt signaling and that this effect is mediated by the cell membrane receptor integrin αvβ3.

Finally yet importantly, given that one of the well-recognized functions of TH is related to energetic metabolism that is tightly associated with cell cycle progression, it can be proposed that the effect of TH on Sertoli cell proliferation may be linked to metabolic regulation. In this context, it might be hypothesized that TH utilize cellular energetic sensors such as AMPK and Sirtuins as mediators of their actions on immature Sertoli cells.

AMPK functions as a key energy-sensing kinase by virtue of its exquisite sensitivity to the cellular AMP/ATP ratio. An increase in the latter ratio promotes AMPK phosphorylation and activation by upstream kinases. Recently, the role of AMPK in cell growth and proliferation has captured attention. It has been demonstrated that AMPK activation causes G1/S phase cell cycle arrest in several cell lines (182, 183) and also that mTORC1 signaling can be downregulated by AMPK (184). Riera et al. (45) have shown that AMPK activation reduces FSH-stimulated Sertoli cell proliferation. Activation of AMPK in FSH-stimulated conditions increases p19INK4d, p27Kip1, and p21Cip1 expression. As mentioned before, the regulation of p21Cip1 and p27Kip1 protein levels in response to TH has previously been related to the cessation of proliferation in FSH-stimulated Sertoli cells (148). Noticeably, TH activate AMPK in several cell lines (185–187). Altogether, the above-mentioned results let us speculate that AMPK activation may be involved in the mechanism of action of TH to regulate the transition of Sertoli cells from the mitotic to the postmitotic state during early postnatal development.

Sirtuins are metabolic sensors that have been implicated in a wide range of cellular processes. The mammalian Sir2 family consists of seven members (SIRT1-7) of NAD^+^ dependent type III histone and protein deacetylases. The most extensive studies of these enzymes were conducted toward functions of SIRT1, which is the founding member of this family. Beyond histone deacetylation, this enzyme also deacetylates many non-histone proteins that are involved in several processes ranging from cell cycle regulation to energy homeostasis. Noteworthy, *Sirt1* KO animals are infertile showing decreased testis size and sperm quality, and this fact is accompanied by Sertoli cell immaturity (188). As a consequence, a physiological relevance of SIRT1 in Sertoli cell maturation has been proposed. It has been shown that SIRT1 is present in immature Sertoli cells at least up to 30-day-old rats and that SIRT1 expression in Sertoli cells decreases with the age of the animal. Additionally, SIRT1 activation markedly decreases proliferation and antagonizes FSH action in immature Sertoli cells. The molecular mechanisms involved in antiproliferative effects of SIRT1 activation were also studied. Activation of SIRT1 decreased cyclin D1 and D2 levels and increased p21Cip1 mRNA levels. SIRT1 activation also decreased c-Myc transcriptional activity (189). Remarkably, TH regulate SIRT1 expression and activity in different experimental models (190–192). SIRT1 activation might also be a molecular mechanism utilized by TH operating in immature Sertoli cells at the time of cessation of proliferation and terminal maturation of this cell type aimed to sustain spermatogenesis. However, experimental evidence for the involvement of AMPK and SIRT1 in the mechanism of action utilized by TH to regulate Sertoli cell proliferation has not been obtained yet.

In summary, studies performed so far have demonstrated that TH have a central role in the cessation of Sertoli cell proliferation and promote Sertoli cell maturation through TRα1 and p43 receptors. The mechanisms participating in these processes involve the regulation of Cx43, c-Myc, p21Cip1, and p27Kip1.

### Estrogens

Estrogens play important roles in the regulation of testis development and spermatogenesis (193, 194). 17β-estradiol (E2) is the pre-dominant and most active estrogen produced from testosterone by aromatase enzyme cytochrome P45019 A1, encoded by the *Cyp19a1* gene (195). In males, E2 is present in low concentrations in blood, but its concentration in semen and the rete testis can reach values even higher than in female serum, suggesting a role for estrogens within the testis (196). Sertoli and Leydig cells, spermatocytes and spermatids express the aromatase enzyme (197, 198). Sertoli cells are the major source of estrogens in immature rats whereas Leydig cells are the main source in adult animals (197, 199). Sertoli cell E2 production is regulated by FSH through the increase of Cyp19a1 expression (200). This response of the Sertoli cells to FSH in terms of aromatase activity and E2 secretion markedly declines with age (201).

Genomic actions of estrogens are mediated by the classical nuclear estrogen receptor alpha (ERα or ESR1) and estrogen receptor beta (ERβ or ESR2). In addition to the genomic actions, rapid signaling events have been described. These latter rapid effects may be mediated by: (a) ERα and ERβ localized at or near the plasma membrane (202), (b) truncated variants of ERα called ERα-46 or ERα-36 (203, 204), and/or (c) G protein-coupled estrogen receptor (GPER or GPR30) (205). The rapid responses include activation of different downstream signaling pathways. It has been suggested that receptor post-translational lipid modifications, such as palmitoylation, can play a role facilitating membrane localization of ER (206).

Regarding ER expression in the testis, *in situ* hybridization and immnohistochemical studies carried out in rats at all ages suggested that ERβ was present in nuclei of Sertoli and Leydig cells, whereas ERα was only present in the interstitial space (207, 208). Years later, in studies performed in rat Sertoli cell cultures and utilizing more sensitive techniques, the presence of ERα was demonstrated (209, 210). Lucas et al. (210) found that ERα protein levels decrease, whereas ERβ protein levels increase in Sertoli cells with the age of the animals. There existed conflicting data regarding GPER expression in human and rodent testis (211–215). A careful study confirmed the presence of GPER in Sertoli cells from immature rats (216). Furthermore, GPER was immunodetected in the endoplasmic reticulum and Golgi apparatus, whereas almost no localization in the plasma membrane was observed (217).

One of the first approaches employed to assess the effect of estrogens on Sertoli cell proliferation consisted of the *in vivo* administration of estrogens to rats. In this regard, estrogen treatment reduced Sertoli cell number when administered during proliferative periods (218). Similarly, results obtained in studies carried out *in vivo* using an aromatase inhibitor and a non-specific ER antagonist (ICI 182,780) suggested that Sertoli cell proliferation diminishes by activation of estrogen receptors (219, 220). As ICI 182,780 is able to antagonize the effect of both ERα and ERβ, the results obtained do not distinguish the type of receptor participating in the observed biological effect.

In order to clarify the role of estrogens on testis development, several models of transgenic mice were studied. Among them are those mice overexpressing aromatase (221), *Cyp19a1* KO (222), *Er*α KO (223–225), and *Er*β KO (225, 226). These transgenic mice were not selective for Sertoli cells, and due to the pleiotropic actions of estrogens, the phenotypes observed could not be straightforwardly attributed to direct actions of estrogens on Sertoli cell proliferation.

Studies performed in isolated Sertoli cells using specific agonists and antagonists of ERα and ERβ shed some light to the role of estrogens in proliferation. Estrogens might regulate both proliferation and cell maturation depending on the ER isoform through which they exert the effect. While estrogens modulate Sertoli cell proliferation through ERα, cell cycle exit, and differentiation involve ERβ. Taking into account that ERα expression decreases while ERβ expression increases with the age of the animals, it was postulated that the ERα/ERβ ratio is physiologically relevant to determine the end of cell proliferation and the start of cell differentiation (210).

Concerning the signaling pathways involved in estrogen action in Sertoli cells, Lucas et al. (210) have shown that the interaction of E2 with ERα promotes cell proliferation through the activation of NFκB in a PI3K- and a ERK1/2-dependent manner and that this is accompanied by cyclin D1 induction. On the other hand, the interaction of E2 with ERβ promotes cell cycle exit and cell maturation through the activation of CREB in a PI3K-dependent manner and this leads to the expression of the Sertoli cell differentiation markers –p27Kip, GATA1, and DMRT1. More recent studies have shown that not only are the classical receptors involved in Sertoli cell proliferation but GPER as well. To this respect, Yang et al. (227) have shown that GPER triggers the activation of Src/PI3K/Akt pathway which is involved in E2-induced Sertoli cell proliferation via regulating the expression of S-phase kinase-associated protein 2 (Skp2). Additional studies destined to evaluate the participation of GPER, either alone or in conjunction with ERs, will be necessary for our overall understanding of estrogen biological function in Sertoli cells.

In summary, investigations performed so far have led to the conclusion that estrogens increase proliferation of Sertoli cells through ERα and GPER. On the other hand, at the end of the proliferative period estrogens promote cessation of proliferation and cell maturation through ERβ.

### Retinoic Acid

It has been recognized for decades that signaling through vitamin A is essential for male reproduction. The biologically active form of vitamin A is retinoic acid (RA), which includes all-trans-RA (atRA), and 9-cis-RA (9-cRA). atRA is synthesized and also stored in lipid droplets in the testis (228–231). atRA content of the testis is practically independent from the plasma levels of this metabolite (232), suggesting that endogenous production of atRA has a vital importance for the maintenance of atRA-dependent processes in the seminiferous tubules. Cavazzini et al. (228) showed that atRA synthesizing activity rises about 5-fold at the time of transition to the non-proliferative phenotype of Sertoli cells and that it continues nearly constant thereafter. On the other hand, Raverdeaua et al. (233) showed that preleptotene spermatocytes may be another source of atRA at the time of meiotic initiation. It is worth mentioning that RA may have biological roles in all testicular cell types including germ cells at different stages of maturation. To this respect, several studies demonstrated that vitamin A deficiency in rats induces a progressive loss of germ cells, ultimately yielding seminiferous tubules that contain only Sertoli cells and premeiotic germ cells (234–237).

The actions of RA are mediated through specific nuclear receptors, the so-called retinoic acid receptors (RAR), which work as ligand-dependent transcription factors and that form heterodimers with retinoic X receptors (RXR). There exist three major subtypes of both, RAR protein (α, β, and γ) and RXR protein (α, β, and γ). Expression of various subtypes of RAR and RXR in Sertoli cells of fetal, neonatal, and adult animals has been demonstrated. Particularly, it has been observed that mitotically active rodent Sertoli cells express RARα and β and RXRα and γ (230, 238–240).

The regulation of Sertoli cell mitosis by atRA was evaluated in *in vitro* studies. Buzzard et al. (148) demonstrated that atRA decreases FSH-stimulated [^3^H]-thymidine and BrdU incorporation in cultures of immature Sertoli cells. Concomitantly, atRA increases p21Cip1 and p27Kip1 expression, proteins that are widely known to be involved in cell cycle arrest and in differentiation of Sertoli cells. Additionally, Nicholls et al. (241) showed that atRA inhibits activin A-stimulated Sertoli cell proliferation. Furthermore, the authors showed that inhibition of cell proliferation is accompanied by a decrease in activin A-stimulated cyclin E1 expression and by an increase in the levels of the cell cycle inhibitor p15INK4. These *in vitro* studies suggest that atRA participates in cessation of Sertoli cell proliferation. As previously mentioned, the cessation of Sertoli cell mitosis is accompanied by the formation of inter-Sertoli cell tight junctions, the main component of the BTB in seminiferous tubules. Noticeably, Nicholls et al. (241) also showed that atRA increases transepithelial electrical resistance (TER), a measurement of tight junctions integrity in Sertoli cell cultures, and promotes plasma membrane localization of the tight junction-related proteins claudin-11 and Tjp1. Altogether, these observations are consistent with an anti-proliferative and pro-differentiative role of RA.

The role of RARs and RXRs proteins in testicular physiology has also been analyzed by utilizing total or selective KO animals. *Rar*α, *Rar*γ, or *Rxr*β null animals have abnormal testicular histology and are infertile, whereas *Rar*β and *Rxr*γ null males are fertile, and their testes and genital tract are histologically normal throughout life (242–247). *Rxr*α null fetuses died *in utero*, thus, its precise role in testicular function could not be defined (248). All these previous studies focused on the germ cell population while no specific attention to possible changes in Sertoli cell physiology was paid. Analyzing selective KO animals, Vernet et al. (249) proposed a possible role of RARα in the regulation of Sertoli cell maturation. The latter authors showed in Sertoli cell-specific *Rar*α-conditional KO mice that there is a marked impairment of Sertoli cell capacity to support germ cell development (249). In addition, Hasegawa and Saga (250) evaluated the impact of the overexpression of a dominant-negative form of the RARα receptor (dn-RARα) in Sertoli cells. They observed that the BTB was disrupted during specific seminiferous tubule stages and postulated that this is partially due to a reduction in occludin expression. A role of RA in BTB function is further supported by the observation of partial disruption of tight junctions in vitamin A deficient rats and in *Rar*α KO mice (251–253).

In summary, results obtained so far are consistent with a role of RA in cessation of proliferation and in maturation of Sertoli cells.

### Opioids

Opioids are also present in the male gonad and are involved in the local control of testicular function. Opioids, such as proopiomelanocortin (POMC), α-melanocyte-stimulating hormone (αMSH), and β-endorphin, are mainly produced in Leydig cells and exert direct paracrine actions on Sertoli cells (254–256). Additionally, high affinity opioid binding sites in Sertoli cells obtained from immature and adult rats have been described (257). Later on three major classes of opioid receptors—mu, delta, and kappa—in Sertoli cells were described (258). As for Sertoli cell proliferation, few studies have focused on this issue. Orth (259), utilizing the opioid receptor blocker naloxone, showed that endogenous opiate-like peptides inhibit the proliferative effects of FSH in fetal rat testis. Moreover, the same research group demonstrated that endorphin suppresses FSH-stimulated proliferation of isolated neonatal Sertoli cells possibly through activation of Gi (260). Sixteen years later, da Silva et al. (261) showed that neonatal treatment with naloxone increases the number of Sertoli cells and daily sperm production per testis in adult animals.

Results available so far are consistent with a negative effect of opioids on FSH-stimulated Sertoli cell proliferation.

## Pharmacological Agents and Xenobiotics May Affect Sertoli Cell Proliferation and Maturation

Epidemiological, clinical, and experimental studies suggest that there are multiple possible causes of the progressive decrease in male reproductive function observed over the past 50 years. Drugs used for the treatment of several pathologies or exposure to xenobiotics in early stages of life may alter testicular function and condition future fertility. Current knowledge of the effects of pharmacological agents and xenobiotics on Sertoli cell proliferation and maturation will be summarized in the following sections.

### Pharmacological Agents

Chemotherapy drugs are the most extensively studied in terms of their possible testicular toxicity. It is known that male fertility is affected by chemotherapy treatment. For example, men treated with alkylating agents show oligospermia or azoospermia as well as alterations in the histology of the testis (262–264). Although the deleterious effects caused by chemotherapy in adults are well-known, mainly attributed to their cytotoxic action on germ cells, little is known about possible effects on the Sertoli cell population. Few studies have addressed the possible gonadotoxic effect of chemotherapeutic agents during childhood and puberty. In fact, it was postulated that chemotherapeutic drugs were less harmful when used in the prepubertal stage because the testis was considered quiescent (265, 266). However, studies with cohorts of patients who received high doses of chemotherapy before puberty clearly show an increased risk of infertility (267, 268). Testis damage might be related to the loss of germ cell population and/or alterations in the Sertoli cell proliferation and maturation processes that take place in the immature testis. The latter hypothesis is under debate and further studies will be necessary to definitively sustain that chemotherapy drugs affect Sertoli cell proliferation. Thirty years ago, it was observed that *in vivo* intratesticular injection of cytosine arabinoside, a chemotherapeutic agent, inhibited Sertoli cell proliferation (4). Additionally, it has been observed that treatment of neonatal rats with a single dose of doxorubicin, beyond promoting apoptosis in germ line stem cells, reduced the rate of increase of Sertoli cells leading to a decrease in the final number of them (269). Tremblay and Delbes (270) have also recently observed that doxorubicin decreases Sertoli cell number in culture. In contrast, Nurmio et al. (271) proposed that the germ cell population is the target of doxorubicin toxicity. As for other drugs, it has been shown in immature Sertoli cells in culture that acrolein—a metabolite of cyclophosphamide—induces cytoskeletal changes and oxidative stress (272). On the other hand, a marked loss of germ cells and no change in the Sertoli cell number were observed in neonatal mice testis treated *in vitro* with cisplatin, doxorubicin or the active metabolite of cyclophosphamide—phosphoramide mustard (273).

Some nucleoside analogs, such as acyclovir and ganciclovir, are used as antiviral agents to treat infections caused by herpes simplex virus, cytomegalovirus, varicella zoster virus, and other viruses. Studies performed in adult animals showed that ganciclovir or acyclovir treatment decreases testis weight and sperm count and increases the number of spermatozoa with abnormalities in head and tail (274, 275). On the other hand, Nihi et al. (276) showed that treatment of pregnant mice with a high dose of ganciclovir decreases the number of gonocytes in fetal testis. In addition, these authors showed that animals that have been exposed *in utero* to ganciclovir present decreased adult testis weight concomitant with an increase in the number of seminiferous tubules with partial or complete absence of germ cells. However, the authors did not observe differences in Sertoli cell number per tubular cross section and suggested that the effects induced *in utero* by ganciclovir may result from direct effects on developing germ cells and/or may be secondary to the dysfunction of Sertoli cells. In support of the latter assumption, Qiu et al. (277) showed in the Sertoli cell line SerW3 that ganciclovir and acyclovir induce a decrease in Cx43 expression, a protein that is essential for proper sertoli cell maturation.

Nonsteroidal antiinflammatory drugs and analgesic drugs, such as ibuprofen—isobutylphenylpropionic acid—and paracetamol—acetyl-p-aminophenol–, are widely used to treat inflammation and pain and commonly prescribed in pregnant women and in children. Just a couple of studies analyzed the effect of these drugs in the Sertoli cell population. Ben Maamar et al. (278) showed that *in vitro* exposure of fetal human testis to ibuprofen does not modify the number of Sertoli cells but decreases AMH and SOX9 expression, suggesting a role in Sertoli cell maturation. Recently, Rossitto et al. (279) showed that treatment of pregnant mice with a combination of paracetamol and ibuprofen promotes a reduction of fetal germ cells mitosis and a decrease in sperm count in offspring's adulthood. These authors also showed that this treatment does not modify fetal or postnatal Sertoli cell proliferation but induces a delay in Sertoli cell maturation. Altogether, these studies support the idea that analgesic drugs may alter Sertoli cell maturation.

Metformin—dimethylbiguanide—is one of the most widely used anti-hyperglycemic agent for treating adult patients with type 2 diabetes. Nowadays, its role as a therapeutic agent is expanding and metformin constitutes the treatment of choice in cases of pregnancy disorders, such as gestational diabetes mellitus or preeclampsia, and also in polycystic ovarian syndrome (280, 281). Considering that metformin can cross the placental barrier, fetuses are exposed to the drug (282). In children, the incidence of both type 2 diabetes and obesity has risen at staggering rates and metformin has started to be used in the pediatric population (283–285). Numerous studies have demonstrated that in addition to its strong antidiabetic properties, metformin shows an anti-proliferative activity in cancer cells (286–288). Despite the latter findings, few studies have analyzed the effect of this drug in non-cancer cells. Particularly in the testis, Tartarin et al. (289) have shown that *in vivo* administration of metformin to pregnant mice reduces the number of Sertoli cells in fetal life and at birth in male offspring. On the other hand, Faure et al. (290) showed that metformin inhibits Sertoli cell proliferation and increases p21Cip1 levels *in vitro* and that treatment of chickens for 3 weeks decreases testis weight and seminiferous tubules diameter. More recently, Rindone et al. (291) showed that metformin decreases FSH-stimulated neonatal rat Sertoli cell proliferation *in vitro*. Concomitantly, a reduction in FSH-stimulated cyclins D1 and D2 and an increase in p21Cip1 expression were observed as a result of metformin treatment. Altogether, these studies support the notion that metformin decreases fetal and postnatal Sertoli cell proliferation.

The mechanism of action through which metformin exerts its effects has not been completely elucidated. On the one hand, it has been shown that in the liver, metformin is able to inhibit the mitochondrial isoform of the enzyme glycerol phosphate dehydrogenase, resulting in a decrease in the levels of dihydroxyacetone phosphate and an increase in the NADH/NAD^+^ ratio in the cytoplasm (292). On the other hand, it has been shown that metformin partially inhibits the complex I of the respiratory chain, which produces a decrease in cellular energy levels that results in the activation of the AMPK (293, 294). As mentioned before, it has been demonstrated that AMPK activation inhibits Sertoli cell proliferation (45). In this context, it has been shown that metformin activates AMPK in mitotically active Sertoli cells (290, 291). In addition, Rindone et al. (291) showed that metformin inhibits FSH-stimulated mTORC1/p70S6K pathway, a signaling pathway that is involved in FSH-stimulation of Sertoli cell proliferation (45). These studies suggest that metformin can counteract the effects of FSH on Sertoli cell proliferation by modulating AMPK and mTORC1/p70S6K pathways. Bearing in mind that metformin is now being used in pregnant women, potentially gaining access to male fetuses, and in children at the same time as Sertoli cells proliferate, attention should be paid to a possible alteration in the final number of Sertoli cells in adulthood.

In summary, experimental evidence for chemotherapy, antiviral, analgesic, and anti-hyperglycemic drugs suggests that they may potentially affect the final number and/or the maturation of Sertoli cells and consequently sperm counts in adulthood. Longitudinal studies evaluating fertility in patients treated for prolonged periods with the above-mentioned drugs would be helpful to gain full confidence in their use at any stage of their lifespan.

### Xenobiotics

Exposure to environmental foreign chemical substances derived from modern life style represents a growing concern due to the impact of these pollutants on developmental and reproductive functions in mammals. Chemical compounds that derive from industrial manufacturing, pesticides and herbicides utilized in agricultural practices, waste accumulation, and burning residues, generally termed xenobiotics, are only a few examples of the contaminants that affect human daily life. Nowadays, it is well-known that testis function is a primary target of a large number of pollutants. Delayed establishment of spermatogenesis (295), impaired differentiation of internal and external male genital structures (296), reduced sperm production and disruption of the hypothalamic–pituitary–gonadal axis (297), decreased anogenital distance and decreased prostate and seminal vesicle weights (298, 299), among others, represent just some of the multiple observations performed.

Several studies have focused on the understanding of the mechanisms involved in the damage of the male reproductive system. Most of the xenobiotics act as endocrine disruptors in multiple organs by mimicking naturally occurring hormones or by binding to hormone receptors (e.g., ER, AR, TR, and so forth) and blocking the action of the hormone. In some cases, xenobiotics induce oxidative stress, decrease activities of antioxidant enzymes and produce perturbation of the tight junctional proteins (300, 301). In the testis, xenobiotics can alter BTB integrity by promoting a loss of gap junction function (302). Environmental toxicants also induce testicular cell apoptosis, and the role of the Fas/FasL signaling pathway has been demonstrated (303, 304). The latter reports represent a brief list of the numerous effects of xenobiotics that have been observed on testicular function. The majority of the studies were performed in adult animals, while there are few investigations on the possible role of xenobiotics in early periods of life when Sertoli cells are proliferating. In the next paragraphs, available information on the effects of toxicants on Sertoli cells during early periods of life is presented.

Phthalic acid esters are widespread in the environment. These compounds are used as plasticizers in food packaging, some children's products and some polyvinyl chloride (PVC) medical devices. *In vitro* experiments show that low levels of mono (2-ethylhexyl) phthalate (MEHP) disrupt Sertoli cell-gonocyte physical interaction and suppress Sertoli cell proliferation (305). In addition, treatment of neonatal rats with low levels of di (2-ethylhexyl) phthalate (DEHP) and its metabolite, MEHP, induces a decrease in Sertoli cell proliferation accompanied by a decrease in cyclin D2 levels (306). More recently, it has been shown that MEHP can disrupt prepubertal Sertoli cell proliferation by increasing intracellular ROS levels (307). On the other hand, it has been shown that another plasticizer of the phthalate family, mono-n-butyl phthalate (MBP), induces immature Sertoli cell proliferation up-regulating ERK1/2 signaling pathway (308).

Bisphenol A (BPA) is a monomer used in the manufacture of polycarbonate plastics and epoxy resins that are present in multitude of consumer products. BPA is an estrogenic endocrine disruptor very well-known for its ubiquitous presence and its effects on male reproduction (309). It has been shown that BPA alters Sertoli cell proliferation positively or negatively in a highly dose dependent manner (310). Additional compounds that can act as endocrine disruptors have been analyzed. It has been shown that oral exposure to the pesticide methoxychlor, a compound with estrogenic/antiandrogenic effects, reduces the number of Sertoli cells. Consistent with the importance of Sertoli cell number on daily sperm production, the study also shows that adult testicular weight and the amount of epididymal spermatozoa are significantly reduced (311). Zearalenone, a mycotoxin that is present in human and animal food and that has estrogenic activity, produces Sertoli cell cycle arrest that is mediated by alterations in PI3K/Akt/mTORC1 signaling (312).

Polychlorinated biphenyls, commonly called PCBs, are mixtures of chlorinated compounds that were used as insulation, coolants, and lubricants in transformers, capacitors, and other electrical equipment. They were also used in plasticizers, surface coatings, inks, adhesives, pesticides, and other products. PCBs cause hypothyroidism in animals (313). Regarding Sertoli cell proliferation, continuous exposure of lactating female rats to PCBs increases testis weight, sperm production, and Sertoli cell number in the adult male offspring. The observed increase in Sertoli cell number has been directly related to the hypothyroidism that these animals present (314, 315).

Our research group is presently investigating a possible deleterious effect of glyphosate, a widely used herbicide in agriculture, on Sertoli cell function. We have observed that glyphosate and particularly its commercial formulation Roundup decreases FSH-stimulated BrdU incorporation in Sertoli cell cultures. A decrease in cyclins and an increase in cell cycle inhibitors expression were also observed, and these results suggest that Roundup may affect Sertoli cell proliferation. *In vivo* studies are currently being performed in order to establish the actual impact of this herbicide in early periods of life.

Finally, gap junctions are potential targets for many environmental compounds. At the gonadal level, by using the Sertoli cell line SerW3, it has been reported that gap junctions and Cx43 expression are particularly altered in response to a large number of xenobiotics, such as toxins, xenoestrogens, pesticides, herbicides, heavy metals, and non-ylphenol (316, 317). The compelling evidence showing that Cx43 is essential for Sertoli and germ cell proliferation, differentiation, and survival and that environmental toxicants can interfere with normal Sertoli cell proliferation by altering Cx43 expression has been extensively reviewed by Pointis et al. (318).

In summary, experimental evidence indicate that Sertoli cell proliferation can also be a process that may be disturbed as a result of the exposure to environmental toxicants.

## Conclusion

**Figure 1:**
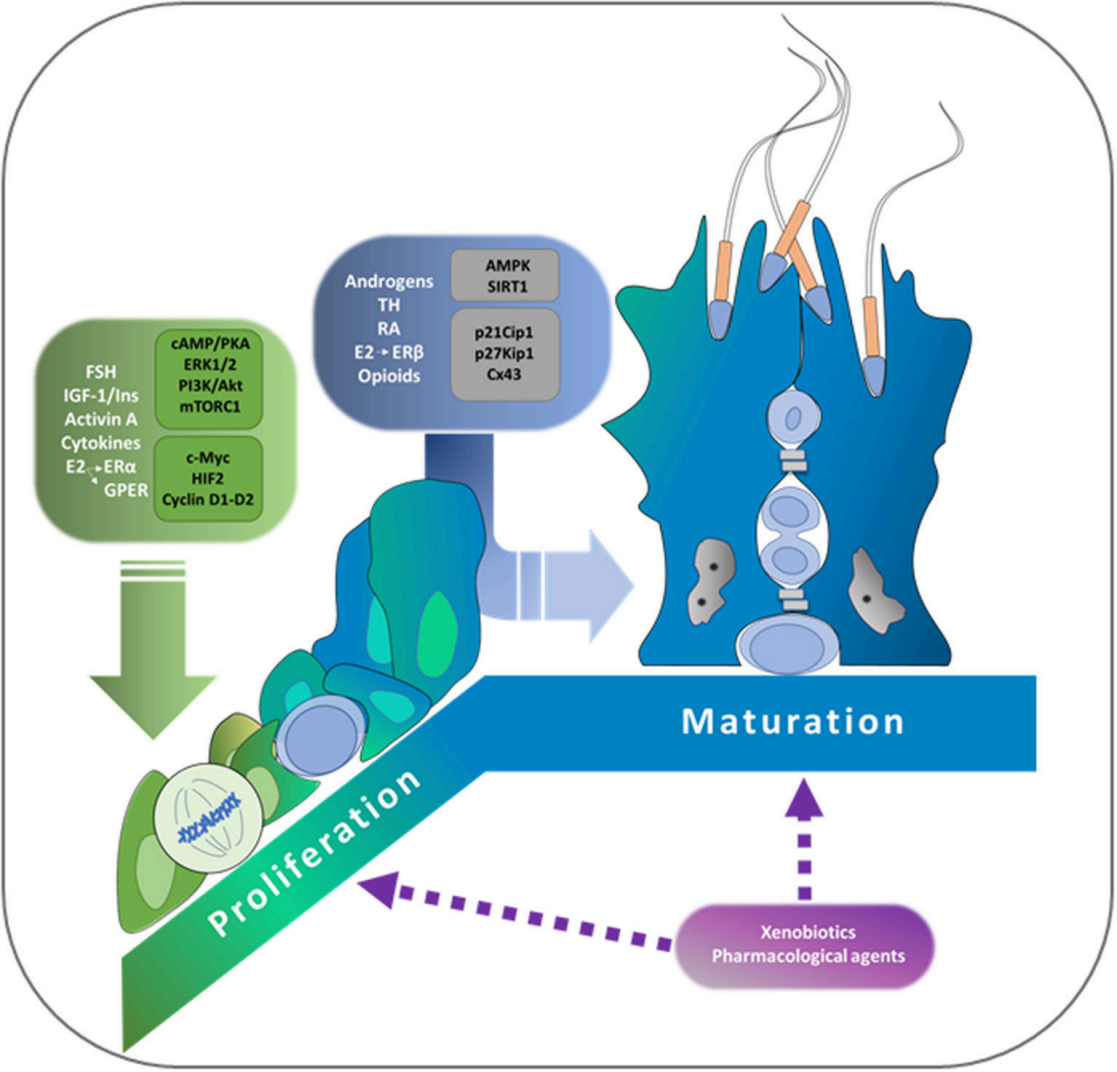
Schematic representation of the main regulators of proliferation and of cessation of proliferation and/or maturation of Sertoli cells. Hormones and paracrine factors that stimulate proliferation are depicted in green and those that promote the cessation of proliferation and/or maturation are depicted in blue. Signal transduction pathways and possible mechanisms involved are also included in the schema. FSH, Follicle-Stimulating Hormone; E2, 17β-estradiol; ERα, Estrogen Receptor α; GPER, G protein-coupled estrogen receptor; IGF-1, Insulin-like growth factor 1; ins, insulin; PI3K, phosphatidyl-inositide-3 kinase; ERK1/2, extracellular regulated kinase 1/2; PKA, cAMP-dependent kinase; mTORC1, mammalian target of rapamycin complex 1; HIF2, Hypoxia inducible factor 2; TH, thyroid hormones; RA, retinoic acid; ERβ, Estrogen Receptor β; AMPK, AMP-activated protein kinase; SIRT1, sirtuin 1; Cx43, connexin 43.

The final number of Sertoli cells reached during the proliferative periods determines both adult testicular size and sperm production capacity in adulthood. This final number of Sertoli cells results from events in fetal, neonatal and peripubertal life. Additionally, terminal differentiation of Sertoli cells, which involves loss of proliferative activity, formation of inter-Sertoli cell tight junctions and establishment of the BTB, is necessary to sustain spermatogenesis. Thus, a perfectly synchronized orchestra involving hormones, signal transduction pathways and molecular mechanisms that play to control Sertoli cell proliferation, and to promote the acquisition of a mature Sertoli cell phenotype is determinant for the future fertility. We have tried to summarize what is known about molecular mechanisms controlling Sertoli cell proliferation and maturation. A few examples of how exposure to environmental toxicants or pharmacological agents in early periods of life can compromise Sertoli cell number are discussed. The topics reviewed are summarized in Figure 1.

## Author Contributions

All authors listed have made a substantial, direct and intellectual contribution to the work, and approved it for publication.

### Conflict of Interest Statement

The authors declare that the research was conducted in the absence of any commercial or financial relationships that could be construed as a potential conflict of interest.

## References

[1] Berndtson WE Igboeli G Pickett BW. Relationship of absolute numbers of Sertoli cells to testicular size and spermatogenesis in young beef bulls. J Anim Sci. (1987) 64:241–6. 10.2527/jas1987.641241x 3818487

[2] Johnson L Zane RS Petty CS Neaves WB. Quantification of the human Sertoli cell population: its distribution, relation to germ cell numbers, and age-related decline. Biol Reprod. (1984) 31:785–95. 10.1095/biolreprod31.4.785 6509142

[3] Griswold MD. Interactions between germ cells and Sertoli cells in the testis. Biol Reprod. (1995) 52:211–6. 10.1095/biolreprod52.2.211 7711190

[4] Orth JM Gunsalus GL Lamperti AA. Evidence from Sertoli cell-depleted rats indicates that spermatid number in adults depends on numbers of Sertoli cells produced during perinatal development. Endocrinology. (1988) 122:787–94. 10.1210/endo-122-3-787 3125042

[5] Sharpe RM McKinnell C Kivlin C Fisher JS. Proliferation and functional maturation of Sertoli cells, and their relevance to disorders of testis function in adulthood. Reproduction. (2003) 125:769–84. 10.1530/rep.0.1250769 12773099

[6] Sertoli E Dell'esistenza di particolari cellule ramificate nei canalicoli seminiferi del testiculo umano. Il Morgagni. (1865) 7:31–9.

[7] Parvinen M Regulation of the seminiferous epithelium. Endocr Rev. (1982) 3:404–17. 10.1210/edrv-3-4-404 6295753

[8] Russell LD Griswold MD The Sertoli Cell. Clearwater, FL: Cache River Press (1993).

[9] Skinner MK Griswold MD Sertoli Cell Biology. Amsterdam: Elsevier (2005).

[10] Clermont Y Perey B. Quantitative study of the cell population of the seminiferous tubules in immature rats. Am J Anatomy. (1957) 100:241–67. 10.1002/aja.1001000205 13435229

[11] Steinberger A Steinberger E. Replication pattern of Sertoli cells in maturing rat testis *in vivo* and in organ culture. Biol Reprod. (1971) 4:84–7. 10.1093/biolreprod/4.1.84 5110903

[12] Griswold MD Solari A Tung PS Fritz IB. Stimulation by follicle-stimulating hormone of DNA synthesis and of mitosis in cultured Sertoli cells prepared from testes of immature rats. Mol Cell Endocrinol. (1977) 7:151–65. 10.1016/0303-7207(77)90064-8 193747

[13] Orth JM. Proliferation of Sertoli cells in fetal and postnatal rats: a quantitative autoradiographic study. Anat Rec. (1982) 203:485–92. 10.1002/ar.1092030408 7137603

[14] Orth JM. The role of follicle-stimulating hormone in controlling Sertoli cell proliferation in testes of fetal rats. Endocrinology. (1984) 115:1248–55. 10.1210/endo-115-4-1248 6090096

[15] Carlsen E Giwercman A Keiding N Skakkebaek NE. Evidence for decreasing quality of semen during past 50 years. BMJ. (1992) 305:609–13. 10.1136/bmj.305.6854.609 1393072 PMC1883354

[16] Aitken RJ. Human spermatozoa: revelations on the road to conception. F1000prime Rep. (2013) 5:39. 10.12703/P5-39 24167720 PMC3790566

[17] Sengupta P Nwagha U Dutta S Krajewska-Kulak E Izuka E. Evidence for decreasing sperm count in African population from 1965 to 2015. Afr Health Sci. (2017) 17:418–27. 10.4314/ahs.v17i2.16 29062337 PMC5637027

[18] Levine H Jorgensen N Martino-Andrade A Mendiola J Weksler-Derri D Mindlis I. Temporal trends in sperm count: a systematic review and meta-regression analysis. Hum Reprod Update. (2017) 23:646–59. 10.1093/humupd/dmx022 28981654 PMC6455044

[19] Jurewicz J Radwan M Sobala W Ligocka D Radwan P Bochenek M. Lifestyle and semen quality: role of modifiable risk factors. Syst Biol Reprod Med. (2014) 60:43–51. 10.3109/19396368.2013.840687 24074254

[20] Knez J. Endocrine-disrupting chemicals and male reproductive health. Reprod Biomed Online. (2013) 26:440–8. 10.1016/j.rbmo.2013.02.005 23510680

[21] Bonde JP. Occupational causes of male infertility. Curr Opin Endocrinol Diab Obes. (2013) 20:234–9. 10.1097/MED.0b013e32835f3d4b 23422246

[22] Sharma R Biedenharn KR Fedor JM Agarwal A. Lifestyle factors and reproductive health: taking control of your fertility. Reprod Biol Endocrinol. (2013) 11:66. 10.1186/1477-7827-11-66 23870423 PMC3717046

[23] Sharpe RM Fetal/neonatal hormones and reproductive function of the male in adulthood. In: Wheeler T Barker DJP O'Brien PMS Royal College of O, Gynaecologists, Programming RSGoF, editors. Fetal Programming: Influences on Development and Disease in Later Life. London: RCOG Press (1999). p. 187–94.

[24] Papkoff H Ekblad M. Ovine follicle stimulating hormone: preparation and characterization of its subunits. Biochem Biophys Res Commun. (1970) 40:614–21. 10.1016/0006-291X(70)90948-4 5492157

[25] Simoni M Gromoll J Nieschlag E. The follicle-stimulating hormone receptor: biochemistry, molecular biology, physiology, and pathophysiology. Endocr Rev. (1997) 18:739–73. 10.1210/er.18.6.739 9408742

[26] Heckert LL Griswold MD. The expression of the follicle-stimulating hormone receptor in spermatogenesis. Recent Prog Horm Res. (2002) 57:129–48. 10.1210/rp.57.1.129 12017540 PMC1496959

[27] Heckert L Griswold MD. Expression of the FSH receptor in the testis. Recent Prog Horm Res. (1993) 48:61–77. 10.1016/B978-0-12-571148-7.50006-3 8441864

[28] Griswold M Mably E Fritz IB. Stimulation by follicle stimulating hormone and dibutyryl cyclic AMP of incorporation of 3H-thymidine into nuclear DNA of cultured Sertoli cell-enriched preparations from immature rats. Curr Top Mol Endocrinol. (1975) 2:413–20. 10.1007/978-1-4613-4440-7_29 195775

[29] Griswold MD Mably ER Fritz IB. FSH stimulation of DNA synthesis in Sertoli cells in culture. Mol Cell Endocrinol. (1976) 4:139–49. 10.1016/0303-7207(76)90033-2 174964

[30] Orth JM Higginbotham CA Salisbury RL Hemicastration causes and testosterone prevents enhanced uptake of [3H] thymidine by Sertoli cells in testes of immature rats. Biol Reprod. (1984) 30:263–70. 10.1095/biolreprod30.1.263 6421336

[31] Almiron I Chemes H. Spermatogenic onset. II. FSH modulates mitotic activity of germ and Sertoli cells in immature rats. Int J Androl. (1988) 11:235–46. 10.1111/j.1365-2605.1988.tb00998.x 3137178

[32] Singh J Handelsman DJ. The effects of recombinant FSH on testosterone-induced spermatogenesis in gonadotrophin-deficient (hpg) mice. J Androl. (1996) 17:382–93. 8889701

[33] Meachem SJ McLachlan RI de Kretser DM Robertson DM Wreford NG. Neonatal exposure of rats to recombinant follicle stimulating hormone increases adult Sertoli and spermatogenic cell numbers. Biol Reprod. (1996) 54:36–44. 10.1095/biolreprod54.1.36 8837998

[34] Haywood M Spaliviero J Jimemez M King NJ Handelsman DJ Allan CM Sertoli and germ cell development in hypogonadal (hpg) mice expressing transgenic follicle-stimulating hormone alone or in combination with testosterone. Endocrinology. (2003) 144:509–17. 10.1210/en.2002-220710 12538611

[35] Allan CM Garcia A Spaliviero J Zhang FP Jimenez M Huhtaniemi I. Complete Sertoli cell proliferation induced by follicle-stimulating hormone (FSH) independently of luteinizing hormone activity: evidence from genetic models of isolated FSH action. Endocrinology. (2004) 145:1587–93. 10.1210/en.2003-1164 14726449

[36] Dierich A Sairam MR Monaco L Fimia GM Gansmuller A LeMeur M. Impairing follicle-stimulating hormone (FSH) signaling *in vivo*: targeted disruption of the FSH receptor leads to aberrant gametogenesis and hormonal imbalance. Proc Natl Acad Sci USA. (1998) 95:13612–7. 10.1073/pnas.95.23.13612 9811848 PMC24867

[37] Abel MH Wootton AN Wilkins V Huhtaniemi I Knight PG Charlton HM. The effect of a null mutation in the follicle-stimulating hormone receptor gene on mouse reproduction. Endocrinology. (2000) 141:1795–803. 10.1210/endo.141.5.7456 10803590

[38] O'Shaughnessy PJ Monteiro A Abel M. Testicular development in mice lacking receptors for follicle stimulating hormone and androgen. PLoS ONE. (2012) 7:e35136. 10.1371/journal.pone.0035136 22514715 PMC3325994

[39] Means AR Huckins C. Coupled events in the early biochemical actions of FSH on the Sertoli cells of the testis. Curr Top Mol Endocrinol. (1974) 1:145–65. 10.1007/978-1-4684-2595-6_8 4377983

[40] Dattatreyamurty B Figgs LW Reichert LE Jr. Physical and functional association of follitropin receptors with cholera toxin-sensitive guanine nucleotide-binding protein. J Biol Chem. (1987) 262:11737–45. 3114250

[41] Gloaguen P Crepieux P Heitzler D Poupon A Reiter E. Mapping the follicle-stimulating hormone-induced signaling networks. Front Endocrinol. (2011) 2:45. 10.3389/fendo.2011.00045 22666216 PMC3364461

[42] Ulloa-Aguirre A Reiter E Crepieux P. FSH receptor signaling: complexity of interactions and signal diversity. Endocrinology. (2018) 159:3020–35. 10.1210/en.2018-00452 29982321

[43] Crepieux P Marion S Martinat N Fafeur V Vern YL Kerboeuf D. The ERK-dependent signalling is stage-specifically modulated by FSH, during primary Sertoli cell maturation. Oncogene. (2001) 20:4696–709. 10.1038/sj.onc.1204632 11498792

[44] Musnier A Heitzler D Boulo T Tesseraud S Durand G Lecureuil C. Developmental regulation of p70 S6 kinase by a G protein-coupled receptor dynamically modelized in primary cells. Cell Mol Life Sci. (2009) 66:3487–503. 10.1007/s00018-009-0134-z 19730801 PMC11115785

[45] Riera MF Regueira M Galardo MN Pellizzari EH Meroni SB Cigorraga SB. Signal transduction pathways in FSH regulation of rat Sertoli cell proliferation. Am J Physiol Endocrinol Metab. (2012) 302:E914–23. 10.1152/ajpendo.00477.2011 22275758

[46] Walker WH Fucci L Habener JF. Expression of the gene encoding transcription factor cyclic adenosine 3',5'-monophosphate (cAMP) response element-binding protein (CREB): regulation by follicle-stimulating hormone-induced cAMP signaling in primary rat Sertoli cells. Endocrinology. (1995) 136:3534–45. 10.1210/endo.136.8.7628390 7628390

[47] Delfino F Walker WH. Stage-specific nuclear expression of NF-kappaB in mammalian testis. Mol Endocrinol. (1998) 12:1696–707. 9817596 10.1210/mend.12.11.0194

[48] Hamil KG Conti M Shimasaki S Hall SH. Follicle-stimulating hormone regulation of AP-1: inhibition of c-jun and stimulation of jun-B gene transcription in the rat Sertoli cell. Mol Cell Endocrinol. (1994) 99:269–77. 10.1016/0303-7207(94)90017-5 8206334

[49] Lim K Hwang BD. Follicle-stimulating hormone transiently induces expression of protooncogene c-myc in primary Sertoli cell cultures of early pubertal and prepubertal rat. Mol Cell Endocrinol. (1995) 111:51–6. 10.1016/0303-7207(95)03543-G 7649352

[50] Galardo MN Gorga A Merlo JP Regueira M Pellizzari EH Cigorraga SB. Participation of HIFs in the regulation of Sertoli cell lactate production. Biochimie. (2017) 132:9–18. 10.1016/j.biochi.2016.10.006 27750035

[51] Gorga A Rindone G Regueira M Riera MF Pellizzari EH Cigorraga SB. HIF involvement in the regulation of rat Sertoli cell proliferation by FSH. Biochem Biophys Res Commun. (2018) 502:508–14. 10.1016/j.bbrc.2018.05.206 29859192

[52] Kondo K Kim WY Lechpammer M Kaelin WG Jr. Inhibition of HIF2alpha is sufficient to suppress pVHL-defective tumor growth. PLoS Biol. (2003) 1:E83. 10.1371/journal.pbio.0000083 14691554 PMC300692

[53] Raval RR Lau KW Tran MG Sowter HM Mandriota SJ Li JL. Contrasting properties of hypoxia-inducible factor 1 (HIF-1) and HIF-2 in von Hippel-Lindau-associated renal cell carcinoma. Mol Cell Biol. (2005) 25:5675–86. 10.1128/MCB.25.13.5675-5686.2005 15964822 PMC1157001

[54] Taniguchi CM Emanuelli B Kahn CR. Critical nodes in signalling pathways: insights into insulin action. Nat Rev Mol Cell Biol. (2006) 7:85–96. 10.1038/nrm1837 16493415

[55] Baker J Hardy MP Zhou J Bondy C Lupu F Bellve AR. Effects of an Igf1 gene null mutation on mouse reproduction. Mol Endocrinol. (1996) 10:903–18. 8813730 10.1210/mend.10.7.8813730

[56] Villalpando I Lira E Medina G Garcia-Garcia E Echeverria O. Insulin-like growth factor 1 is expressed in mouse developing testis and regulates somatic cell proliferation. Exp Biol Med. (2008) 233:419–26. 10.3181/0708-RM-212 18367630

[57] Borland K Mita M Oppenheimer CL Blinderman LA Massague J Hall PF. The actions of insulin-like growth factors I and II on cultured Sertoli cells. Endocrinology. (1984) 114:240–6. 10.1210/endo-114-1-240 6360662

[58] Oonk RB Grootegoed JA. Insulin-like growth factor I (IGF-I) receptors on Sertoli cells from immature rats and age-dependent testicular binding of IGF-I and insulin. Mol Cell Endocrinol. (1988) 55:33–43. 10.1016/0303-7207(88)90088-3 2966084

[59] Jaillard C Chatelain PG Saez JM. *In vitro* regulation of pig Sertoli cell growth and function: effects of fibroblast growth factor and somatomedin-C. Biol Reprod. (1987) 37:665–74. 10.1095/biolreprod37.3.665 3118982

[60] Khan SA Ndjountche L Pratchard L Spicer LJ Davis JS. Follicle-stimulating hormone amplifies insulin-like growth factor I-mediated activation of AKT/protein kinase B signaling in immature rat Sertoli cells. Endocrinology. (2002) 143:2259–67. 10.1210/endo.143.6.8838 12021190

[61] Dance A Thundathil J Blondin P Kastelic J. Enhanced early-life nutrition of Holstein bulls increases sperm production potential without decreasing postpubertal semen quality. Theriogenology. (2016) 86:687–94 e2. 10.1016/j.theriogenology.2016.02.022 27114168

[62] Froment P Vigier M Negre D Fontaine I Beghelli J Cosset FL. Inactivation of the IGF-I receptor gene in primary Sertoli cells highlights the autocrine effects of IGF-I. J Endocrinol. (2007) 194:557–68. 10.1677/JOE-07-0258 17761895

[63] Saez JM Chatelain PG Perrard-Sapori MH Jaillard C Naville D. Differentiating effects of somatomedin-C/insulin-like growth factor I and insulin on Leydig and Sertoli cell functions. Reprod Nutr Dev. (1988) 28:989–1008. 10.1051/rnd:19880701 2854290

[64] Pitetti JL Calvel P Zimmermann C Conne B Papaioannou MD Aubry F. An essential role for insulin and IGF1 receptors in regulating sertoli cell proliferation, testis size, and FSH action in mice. Mol Endocrinol. (2013) 27:814–27. 10.1210/me.2012-1258 23518924 PMC5416760

[65] Griffeth RJ Carretero J Burks DJ. Insulin receptor substrate 2 is required for testicular development. PLoS ONE. (2013) 8:e62103. 10.1371/journal.pone.0062103 23741292 PMC3669358

[66] Naville D Chatelain PG Avallet O Saez JM Control of production of insulin-like growth factor I by pig Leydig and Sertoli cells cultured alone or together. Cell-cell interactions. Mol Cell Endocrinol. (1990) 70:217–24. 10.1016/0303-7207(90)90212-Q 2113875

[67] Yamamoto T Nakayama Y Abe SI. Mammalian follicle-stimulating hormone and insulin-like growth factor I (IGF-I) up-regulate IGF-I gene expression in organ culture of newt testis. Mol Reprod Dev. (2001) 60:56–64. 10.1002/mrd.1061 11550268

[68] Smith EP Dickson BA Chernausek SD. Insulin-like growth factor binding protein-3 secretion from cultured rat sertoli cells: dual regulation by follicle stimulating hormone and insulin-like growth factor-I. Endocrinology. (1990) 127:2744–51. 10.1210/endo-127-6-2744 1701126

[69] Rappaport MS Smith EP. Insulin-like growth factor (IGF) binding protein 3 in the rat testis: follicle-stimulating hormone dependence of mRNA expression and inhibition of IGF-I action on cultured Sertoli cells. Biol Reprod. (1995) 52:419–25. 10.1095/biolreprod52.2.419 7536052

[70] Abel MH Baker PJ Charlton HM Monteiro A Verhoeven G De Gendt K. Spermatogenesis and sertoli cell activity in mice lacking sertoli cell receptors for follicle-stimulating hormone and androgen. Endocrinology. (2008) 149:3279–85. 10.1210/en.2008-0086 18403489 PMC2592075

[71] Sherwood OD. Relaxin's physiological roles and other diverse actions. Endocr Rev. (2004) 25:205–34. 10.1210/er.2003-0013 15082520

[72] Dschietzig T Bartsch C Baumann G Stangl K. Relaxin-a pleiotropic hormone and its emerging role for experimental and clinical therapeutics. Pharmacol Ther. (2006) 112:38–56. 10.1016/j.pharmthera.2006.03.004 16647137

[73] Hsu SY Nakabayashi K Nishi S Kumagai J Kudo M Sherwood OD. Activation of orphan receptors by the hormone relaxin. Science. (2002) 295:671–4. 10.1126/science.1065654 11809971

[74] Cardoso LC Nascimento AR Royer C Porto CS Lazari MF. Locally produced relaxin may affect testis and vas deferens function in rats. Reproduction. (2010) 139:185–96. 10.1530/REP-09-0146 19812235

[75] Filonzi M Cardoso LC Pimenta MT Queiroz DB Avellar MC Porto CS. Relaxin family peptide receptors Rxfp1 and Rxfp2: mapping of the mRNA and protein distribution in the reproductive tract of the male rat. Reprod Biol Endocrinol. (2007) 5:29. 10.1186/1477-7827-5-29 17623071 PMC1947996

[76] Nascimento AR Pimenta MT Lucas TF Royer C Porto CS Lazari MF. Intracellular signaling pathways involved in the relaxin-induced proliferation of rat Sertoli cells. Eur J Pharmacol. (2012) 691:283–91. 10.1016/j.ejphar.2012.07.021 22819701

[77] Samuel CS Zhao C Yang Q Wang H Tian H Tregear GW. The relaxin gene knockout mouse: a model of progressive scleroderma. J Invest Dermatol. (2005) 125:692–9. 10.1111/j.0022-202X.2005.23880.x 16185267

[78] Nascimento AR Pimenta MT Lucas TF Porto CS Lazari MF. Relaxin and Sertoli cell proliferation. Ital J Anat Embryol. (2013) 118 (1 Suppl.):26–8. 10.13128/IJAE-13885 24640565

[79] Nascimento AR Macheroni C Lucas TF Porto CS Lazari MF. Crosstalk between FSH and relaxin at the end of the proliferative stage of rat Sertoli cells. Reproduction. (2016) 152:613–28. 10.1530/REP-16-0330 27601715

[80] Franchimont P Demoulin A Verstraelen-Proyard J Hazee-Hagelstein MT Tunbridge WM Identification in human seminal fluid of an inhibin-like factor which selectively regulates FSH secretion. J Reprod Fertil Suppl. (1979) 26:123–33. 293406

[81] Ling N Ying SY Ueno N Shimasaki S Esch F Hotta M. A homodimer of the beta-subunits of inhibin A stimulates the secretion of pituitary follicle stimulating hormone. Biochem Biophys Res Commun. (1986) 138:1129–37. 10.1016/S0006-291X(86)80400-4 3092817

[82] Barton DE Yang-Feng TL Mason AJ Seeburg PH Francke U. Mapping of genes for inhibin subunits alpha, beta A, and beta B on human and mouse chromosomes and studies of jsd mice. Genomics. (1989) 5:91–9. 10.1016/0888-7543(89)90091-8 2767687

[83] Hotten G Neidhardt H Schneider C Pohl J Cloning of a new member of the TGF-beta family: a putative new activin beta C chain. Biochem Biophys Res Commun. (1995) 206:608–13. 10.1006/bbrc.1995.1086 7826378

[84] Oda S Nishimatsu S Murakami K Ueno N. Molecular cloning and functional analysis of a new activin beta subunit: a dorsal mesoderm-inducing activity in Xenopus. Biochem Biophys Res Commun. (1995) 210:581–8. 10.1006/bbrc.1995.1699 7755637

[85] Fang J Yin W Smiley E Wang SQ Bonadio J. Molecular cloning of the mouse activin beta E subunit gene. Biochem Biophys Res Commun. (1996) 228:669–74. 10.1006/bbrc.1996.1715 8941337

[86] Mason AJ Hayflick JS Ling N Esch F Ueno N Ying SY. Complementary DNA sequences of ovarian follicular fluid inhibin show precursor structure and homology with transforming growth factor-beta. Nature. (1985) 318:659–63. 10.1038/318659a0 2417121

[87] Roberts V Meunier H Sawchenko PE Vale W. Differential production and regulation of inhibin subunits in rat testicular cell types. Endocrinology. (1989) 125:2350–9. 10.1210/endo-125-5-2350 2676482

[88] de Winter JP Vanderstichele HM Verhoeven G Timmerman MA Wesseling JG de Jong FH. Peritubular myoid cells from immature rat testes secrete activin-A and express activin receptor type II *in vitro*. Endocrinology. (1994) 135:759–67. 10.1210/endo.135.2.8033824 8033824

[89] Buzzard JJ Farnworth PG De Kretser DM O'Connor AE Wreford NG Morrison JR. Proliferative phase sertoli cells display a developmentally regulated response to activin *in vitro*. Endocrinology. (2003) 144:474–83. 10.1210/en.2002-220595 12538607

[90] Meehan T Schlatt S O'Bryan MK de Kretser DM Loveland KL. Regulation of germ cell and Sertoli cell development by activin, follistatin, and FSH. Dev Biol. (2000) 220:225–37. 10.1006/dbio.2000.9625 10753512

[91] Jeanes A Wilhelm D Wilson MJ Bowles J McClive PJ Sinclair AH. Evaluation of candidate markers for the peritubular myoid cell lineage in the developing mouse testis. Reproduction. (2005) 130:509–16. 10.1530/rep.1.00718 16183868

[92] Anderson RA Cambray N Hartley PS McNeilly AS. Expression and localization of inhibin alpha, inhibin/activin betaA and betaB and the activin type II and inhibin beta-glycan receptors in the developing human testis. Reproduction. (2002) 123:779–88. 10.1530/rep.0.1230779 12052232

[93] Archambeault DR Yao HH. Activin A, a product of fetal Leydig cells, is a unique paracrine regulator of Sertoli cell proliferation and fetal testis cord expansion. Proc Natl Acad Sci USA. (2010) 107:10526–31. 10.1073/pnas.1000318107 20498064 PMC2890803

[94] Barakat B O'Connor AE Gold E de Kretser DM Loveland KL. Inhibin, activin, follistatin and FSH serum levels and testicular production are highly modulated during the first spermatogenic wave in mice. Reproduction. (2008) 136:345–59. 10.1530/REP-08-0140 18515316

[95] Fragale A Puglisi R Morena AR Stefanini M Boitani C. Age-dependent activin receptor expression pinpoints activin A as a physiological regulator of rat Sertoli cell proliferation. Mol Hum Reprod. (2001) 7:1107–14. 10.1093/molehr/7.12.1107 11719587

[96] Boitani C Stefanini M Fragale A Morena AR. Activin stimulates Sertoli cell proliferation in a defined period of rat testis development. Endocrinology. (1995) 136:5438–44. 10.1210/endo.136.12.7588293 7588293

[97] Mendis SH Meachem SJ Sarraj MA Loveland KL. Activin A balances Sertoli and germ cell proliferation in the fetal mouse testis. Biol Reprod. (2011) 84:379–91. 10.1095/biolreprod.110.086231 20926807

[98] Archambeault DR Tomaszewski J Childs AJ Anderson RA Yao HH Testicular somatic cells, not gonocytes, are the major source of functional activin A during testis morphogenesis. Endocrinology. (2011) 152:4358–67. 10.1210/en.2011-1288 21952240 PMC3199008

[99] Matzuk MM Kumar TR Bradley A Different phenotypes for mice deficient in either activins or activin receptor type II. Nature. (1995) 374:356–60. 10.1038/374356a0 7885474

[100] Miles DC Wakeling SI Stringer JM van den Bergen JA Wilhelm D Sinclair AH. Signaling through the TGF beta-activin receptors ALK4/5/7 regulates testis formation and male germ cell development. PLoS ONE. (2013) 8:e54606. 10.1371/journal.pone.0054606 23342175 PMC3546992

[101] Itman C Small C Griswold M Nagaraja AK Matzuk MM Brown CW. Developmentally regulated SMAD2 and SMAD3 utilization directs activin signaling outcomes. Dev Dynam. (2009) 238:1688–700. 10.1002/dvdy.21995 19517569 PMC2819023

[102] Nicholls PK Stanton PG Chen JL Olcorn JS Haverfield JT Qian H. Activin signaling regulates Sertoli cell differentiation and function. Endocrinology. (2012) 153:6065–77. 10.1210/en.2012-1821 23117933

[103] Schrewe H Gendron-Maguire M Harbison ML Gridley T. Mice homozygous for a null mutation of activin beta B are viable and fertile. Mechan Dev. (1994) 47:43–51. 10.1016/0925-4773(94)90094-9 7947320

[104] Anawalt BD Bebb RA Matsumoto AM Groome NP Illingworth PJ McNeilly AS. Serum inhibin B levels reflect Sertoli cell function in normal men and men with testicular dysfunction. J Clin Endocrinol Metab. (1996) 81:3341–5. 8784094 10.1210/jcem.81.9.8784094

[105] Matzuk MM Finegold MJ Su JG Hsueh AJ Bradley A. Alpha-inhibin is a tumour-suppressor gene with gonadal specificity in mice. Nature. (1992) 360:313–9. 10.1038/360313a0 1448148

[106] Matzuk MM Finegold MJ Mather JP Krummen L Lu H Bradley A. Development of cancer cachexia-like syndrome and adrenal tumors in inhibin-deficient mice. Proc Natl Acad Sci USA. (1994) 91:8817–21. 10.1073/pnas.91.19.8817 8090730 PMC44697

[107] Li Q Graff JM O'Connor AE Loveland KL Matzuk MM. SMAD3 regulates gonadal tumorigenesis. Mol Endocrinol. (2007) 21:2472–86. 10.1210/me.2007-0147 17595316

[108] Looyenga BD Hammer GD. Genetic removal of Smad3 from inhibin-null mice attenuates tumor progression by uncoupling extracellular mitogenic signals from the cell cycle machinery. Mol Endocrinol. (2007) 21:2440–57. 10.1210/me.2006-0402 17652186

[109] Gnessi L Fabbri A Spera G. Gonadal peptides as mediators of development and functional control of the testis: an integrated system with hormones and local environment. Endocr Rev. (1997) 18:541–609. 10.1210/er.18.4.541 9267764

[110] Loveland KL Klein B Pueschl D Indumathy S Bergmann M Loveland BE. Cytokines in male fertility and reproductive pathologies: immunoregulation and beyond. Front Endocrinol. (2017) 8:307. 10.3389/fendo.2017.00307 29250030 PMC5715375

[111] Gerard N Syed V Bardin W Genetet N Jegou B. Sertoli cells are the site of interleukin-1 alpha synthesis in rat testis. Mol Cell Endocrinol. (1991) 82:R13–6. 10.1016/0303-7207(91)90019-O 1761160

[112] Wang DL Nagpal ML Calkins JH Chang WW Sigel MM Lin T. Interleukin-1 beta induces interleukin-1 alpha messenger ribonucleic acid expression in primary cultures of Leydig cells. Endocrinology. (1991) 129:2862–6. 10.1210/endo-129-6-2862 1954872

[113] Lin T Wang D Nagpal ML. Human chorionic gonadotropin induces interleukin-1 gene expression in rat Leydig cells *in vivo*. Mol Cell Endocrinol. (1993) 95:139–45. 10.1016/0303-7207(93)90039-M 8243804

[114] Hayes R Chalmers SA Nikolic-Paterson DJ Atkins RC Hedger MP. Secretion of bioactive interleukin 1 by rat testicular macrophages *in vitro*. J Androl. (1996) 17:41–9. 8833740

[115] Hayrabedyan S Todorova K Jabeen A Metodieva G Toshkov S Metodiev MV. Sertoli cells have a functional NALP3 inflammasome that can modulate autophagy and cytokine production. Sci Rep. (2016) 6:18896. 10.1038/srep18896 26744177 PMC4705529

[116] Cudicini C Lejeune H Gomez E Bosmans E Ballet F Saez J. Human Leydig cells and Sertoli cells are producers of interleukins-1 and−6. J Clin Endocrinol Metab. (1997) 82:1426–33. 10.1210/jc.82.5.1426 9141528

[117] Gomez E Morel G Cavalier A Lienard MO Haour F Courtens JL. Type I and type II interleukin-1 receptor expression in rat, mouse, and human testes. Biol Reprod. (1997) 56:1513–26. 10.1095/biolreprod56.6.1513 9166705

[118] Petersen C Boitani C Froysa B Soder O. Interleukin-1 is a potent growth factor for immature rat sertoli cells. Mol Cell Endocrinol. (2002) 186:37–47. 10.1016/S0303-7207(01)00680-3 11850120

[119] Petersen C Svechnikov K Froysa B Soder O. The p38 MAPK pathway mediates interleukin-1-induced Sertoli cell proliferation. Cytokine. (2005) 32:51–9. 10.1016/j.cyto.2005.07.014 16181786

[120] Jonsson CK Zetterstrom RH Holst M Parvinen M Soder O. Constitutive expression of interleukin-1alpha messenger ribonucleic acid in rat Sertoli cells is dependent upon interaction with germ cells. Endocrinology. (1999) 140:3755–61. 10.1210/endo.140.8.6900 10433236

[121] De SK Chen HL Pace JL Hunt JS Terranova PF Enders GC. Expression of tumor necrosis factor-alpha in mouse spermatogenic cells. Endocrinology. (1993) 133:389–96. 10.1210/endo.133.1.8319585 8319585

[122] Mauduit C Besset V Caussanel V Benahmed M. Tumor necrosis factor alpha receptor p55 is under hormonal (follicle-stimulating hormone) control in testicular Sertoli cells. Biochem Biophys Res Commun. (1996) 224:631–7. 10.1006/bbrc.1996.1077 8713100

[123] Petersen C Froysa B Soder O. Endotoxin and proinflammatory cytokines modulate Sertoli cell proliferation *in vitro*. J Reprod Immunol. (2004) 61:13–30. 10.1016/j.jri.2003.10.003 15027475

[124] Sharpe RM Regulation of Spermatogenesis. Knobil E Neill JD, editors. New York, NY: Raven Press (1994).

[125] McLachlan RI O'Donnell L Meachem SJ Stanton PG de Kretser DM Pratis K. Identification of specific sites of hormonal regulation in spermatogenesis in rats, monkeys, and man. Recent Prog Horm Res. (2002) 57:149–79. 10.1210/rp.57.1.149 12017541

[126] Griswold SL Behringer RR. Fetal Leydig cell origin and development. Sex Dev. (2009) 3:1–15. 10.1159/000200077 19339813 PMC4021856

[127] Corbier P Edwards DA Roffi J. The neonatal testosterone surge: a comparative study. Arch Int Physiol Biochim Biophys. (1992) 100:127–31. 10.3109/13813459209035274 1379488

[128] Ketelslegers JM Hetzel WD Sherins RJ Catt KJ. Developmental changes in testicular gonadotropin receptors: plasma gonadotropins and plasma testosterone in the rat. Endocrinology. (1978) 103:212–22. 10.1210/endo-103-1-212 217637

[129] Forest MG Cathiard AM. Pattern of plasma testosterone and delta4-androstenedione in normal newborns: evidence for testicular activity at birth. J Clin Endocrinol Metab. (1975) 41:977–80. 10.1210/jcem-41-5-977 1184729

[130] Heemers HV Tindall DJ. Androgen receptor (AR) coregulators: a diversity of functions converging on and regulating the AR transcriptional complex. Endocr Rev. (2007) 28:778–808. 10.1210/er.2007-0019 17940184

[131] Bremner WJ Millar MR Sharpe RM Saunders PT. Immunohistochemical localization of androgen receptors in the rat testis: evidence for stage-dependent expression and regulation by androgens. Endocrinology. (1994) 135:1227–34. 10.1210/endo.135.3.8070367 8070367

[132] Grootegoed JA Peters MJ Mulder E Rommerts FF Van der Molen HJ. Absence of a nuclear androgen receptor in isolated germinal cells of rat testis. Mol Cell Endocrinol. (1977) 9:159–67. 10.1016/0303-7207(77)90117-4 598614

[133] Anthony CT Kovacs WJ Skinner MK. Analysis of the androgen receptor in isolated testicular cell types with a microassay that uses an affinity ligand. Endocrinology. (1989) 125:2628–35. 10.1210/endo-125-5-2628 2792002

[134] Pelletier G Labrie C Labrie F. Localization of oestrogen receptor alpha, oestrogen receptor beta and androgen receptors in the rat reproductive organs. J Endocrinol. (2000) 165:359–70. 10.1677/joe.0.1650359 10810300

[135] Vornberger W Prins G Musto NA Suarez-Quian CA. Androgen receptor distribution in rat testis: new implications for androgen regulation of spermatogenesis. Endocrinology. (1994) 134:2307–16. 10.1210/endo.134.5.8156934 8156934

[136] Zhou X Kudo A Kawakami H Hirano H. Immunohistochemical localization of androgen receptor in mouse testicular germ cells during fetal and postnatal development. Anat Rec. (1996) 245:509–18. 10.1002/(SICI)1097-0185(199607)245:3<509::AID-AR7>3.0.CO;2-M 8800409

[137] Merlet J Racine C Moreau E Moreno SG Habert R. Male fetal germ cells are targets for androgens that physiologically inhibit their proliferation. Proc Natl Acad Sci USA. (2007) 104:3615–20. 10.1073/pnas.0611421104 17360691 PMC1805536

[138] Buzek SW Sanborn BM. Increase in testicular androgen receptor during sexual maturation in the rat. Biol Reprod. (1988) 39:39–49. 10.1095/biolreprod39.1.39 3207797

[139] You L Sar M. Androgen receptor expression in the testes and epididymides of prenatal and postnatal Sprague-Dawley rats. Endocrine. (1998) 9:253–61. 10.1385/ENDO:9:3:253 10221590

[140] Zhou Q Shima JE Nie R Friel PJ Griswold MD. Androgen-regulated transcripts in the neonatal mouse testis as determined through microarray analysis. Biol Reprod. (2005) 72:1010–9. 10.1095/biolreprod.104.035915 15601916

[141] Johnston H Baker PJ Abel M Charlton HM Jackson G Fleming L. Regulation of Sertoli cell number and activity by follicle-stimulating hormone and androgen during postnatal development in the mouse. Endocrinology. (2004) 145:318–29. 10.1210/en.2003-1055 14551232

[142] Atanassova NN Walker M McKinnell C Fisher JS Sharpe RM. Evidence that androgens and oestrogens, as well as follicle-stimulating hormone, can alter Sertoli cell number in the neonatal rat. J Endocrinol. (2005) 184:107–17. 10.1677/joe.1.05884 15642788

[143] Tan KA De Gendt K Atanassova N Walker M Sharpe RM Saunders PT The role of androgens in sertoli cell proliferation and functional maturation: studies in mice with total or Sertoli cell-selective ablation of the androgen receptor. Endocrinology. (2005) 146:2674–83. 10.1210/en.2004-1630 15761038

[144] Skinner MK Fritz IB. Testicular peritubular cells secrete a protein under androgen control that modulates Sertoli cell functions. Proc Natl Acad Sci USA. (1985) 82:114–8. 10.1073/pnas.82.1.114 3855533 PMC396982

[145] Swinnen K Cailleau J Heyns W Verhoeven G. Prostatic stromal cells and testicular peritubular cells produce similar paracrine mediators of androgen action. Endocrinology. (1990) 126:142–50. 10.1210/endo-126-1-142 2403517

[146] Zhang C Yeh S Chen YT Wu CC Chuang KH Lin HY. Oligozoospermia with normal fertility in male mice lacking the androgen receptor in testis peritubular myoid cells. Proc Natl Acad Sci USA. (2006) 103:17718–23. 10.1073/pnas.0608556103 17095600 PMC1693813

[147] Hazra R Corcoran L Robson M McTavish KJ Upton D Handelsman DJ. Temporal role of Sertoli cell androgen receptor expression in spermatogenic development. Mol Endocrinol. (2013) 27:12–24. 10.1210/me.2012-1219 23160479 PMC5416944

[148] Buzzard JJ Wreford NG Morrison JR. Thyroid hormone, retinoic acid, and testosterone suppress proliferation and induce markers of differentiation in cultured rat sertoli cells. Endocrinology. (2003) 144:3722–31. 10.1210/en.2003-0379 12933640

[149] Fix C Jordan C Cano P Walker WH. Testosterone activates mitogen-activated protein kinase and the cAMP response element binding protein transcription factor in Sertoli cells. Proc Natl Acad Sci USA. (2004) 101:10919–24. 10.1073/pnas.0404278101 15263086 PMC503720

[150] Cheng J Watkins SC Walker WH. Testosterone activates mitogen-activated protein kinase via Src kinase and the epidermal growth factor receptor in sertoli cells. Endocrinology. (2007) 148:2066–74. 10.1210/en.2006-1465 17272394

[151] Berg AH Rice CD Rahman MS Dong J Thomas P. Identification and characterization of membrane androgen receptors in the ZIP9 zinc transporter subfamily: I. Discovery in female atlantic croaker and evidence ZIP9 mediates testosterone-induced apoptosis of ovarian follicle cells. Endocrinology. (2014) 155:4237–49. 10.1210/en.2014-1198 25014354 PMC4197986

[152] Bulldan A Dietze R Shihan M Scheiner-Bobis G. Non-classical testosterone signaling mediated through ZIP9 stimulates claudin expression and tight junction formation in Sertoli cells. Cell Signal. (2016) 28:1075–85. 10.1016/j.cellsig.2016.04.015 27164415

[153] Harvey CB Williams GR. Mechanism of thyroid hormone action. Thyroid. (2002) 12:441–6. 10.1089/105072502760143791 12165104

[154] Yen PM. Physiological and molecular basis of thyroid hormone action. Physiol Rev. (2001) 81:1097–142. 10.1152/physrev.2001.81.3.1097 11427693

[155] Wrutniak C Cassar-Malek I Marchal S Rascle A Heusser S Keller JM. A 43-kDa protein related to c-Erb A alpha 1 is located in the mitochondrial matrix of rat liver. J Biol Chem. (1995) 270:16347–54. 10.1074/jbc.270.27.16347 7608204

[156] Casas F Rochard P Rodier A Cassar-Malek I Marchal-Victorion S Wiesner RJ. A variant form of the nuclear triiodothyronine receptor c-ErbAalpha1 plays a direct role in regulation of mitochondrial RNA synthesis. Mol Cell Biol. (1999) 19:7913–24. 10.1128/MCB.19.12.7913 10567517 PMC84876

[157] Bergh JJ Lin HY Lansing L Mohamed SN Davis FB Mousa S. Integrin alphaVbeta3 contains a cell surface receptor site for thyroid hormone that is linked to activation of mitogen-activated protein kinase and induction of angiogenesis. Endocrinology. (2005) 146:2864–71. 10.1210/en.2005-0102 15802494

[158] Buzzard JJ Morrison JR O'Bryan MK Song Q Wreford NG. Developmental expression of thyroid hormone receptors in the rat testis. Biol Reprod. (2000) 62:664–9. 10.1095/biolreprod62.3.664 10684808

[159] Palmero S Trucchi P Prati M Fugassa E Lanni A Goglia F. Effect of thyroid status on the oxidative capacity of Sertoli cells isolated from immature rat testis. Eur J Endocrinol. (1994) 130:308–12. 10.1530/eje.0.1300308 8156106

[160] Zanatta AP Zanatta L Goncalves R Zamoner A Silva FR Rapid responses to reverse T(3) hormone in immature rat Sertoli cells: calcium uptake and exocytosis mediated by integrin. PLoS ONE. (2013) 8:e77176 10.1371/journal.pone.0077176 24130850 PMC3795021

[161] Cooke PS Meisami E Early hypothyroidism in rats causes increased adult testis and reproductive organ size but does not change testosterone levels. Endocrinology. (1991) 129:237–43. 10.1210/endo-129-1-237 2055186

[162] De Franca LR Hess RA Cooke PS Russell LD. Neonatal hypothyroidism causes delayed Sertoli cell maturation in rats treated with propylthiouracil: evidence that the Sertoli cell controls testis growth. Anat Rec. (1995) 242:57–69. 10.1002/ar.1092420108 7604982

[163] van Haaster LH de Jong FH Docter R de Rooij DG. High neonatal triiodothyronine levels reduce the period of Sertoli cell proliferation and accelerate tubular lumen formation in the rat testis, and increase serum inhibin levels. Endocrinology. (1993) 133:755–60. 10.1210/endo.133.2.8344214 8344214

[164] Auharek SA de Franca LR. Postnatal testis development, Sertoli cell proliferation and number of different spermatogonial types in C57BL/6J mice made transiently hypo- and hyperthyroidic during the neonatal period. J Anat. (2010) 216:577–88. 10.1111/j.1469-7580.2010.01219.x 20525087 PMC2871994

[165] Cooke PS Zhao YD Bunick D. Triiodothyronine inhibits proliferation and stimulates differentiation of cultured neonatal Sertoli cells: possible mechanism for increased adult testis weight and sperm production induced by neonatal goitrogen treatment. Biol Reprod. (1994) 51:1000–5. 10.1095/biolreprod51.5.1000 7531505

[166] Palmero S Prati M Bolla F Fugassa E. Tri-iodothyronine directly affects rat Sertoli cell proliferation and differentiation. J Endocrinol. (1995) 145:355–62. 10.1677/joe.0.1450355 7616169

[167] Holsberger DR Buchold GM Leal MC Kiesewetter SE O'Brien DA Hess RA. Cell-cycle inhibitors p27Kip1 and p21Cip1 regulate murine Sertoli cell proliferation. Biol Reprod. (2005) 72:1429–36. 10.1095/biolreprod.105.040386 15728790

[168] Arambepola NK Bunick D Cooke PS. Thyroid hormone and follicle-stimulating hormone regulate Mullerian-inhibiting substance messenger ribonucleic acid expression in cultured neonatal rat Sertoli cells. Endocrinology. (1998) 139:4489–95. 10.1210/endo.139.11.6315 9794457

[169] Arambepola NK Bunick D Cooke PS. Thyroid hormone effects on androgen receptor messenger RNA expression in rat Sertoli and peritubular cells. J Endocrinol. (1998) 156:43–50. 10.1677/joe.0.1560043 9496232

[170] Palmero S De Marco P Fugassa E. Thyroid hormone receptor beta mRNA expression in Sertoli cells isolated from prepubertal testis. J Mol Endocrinol. (1995) 14:131–4. 10.1677/jme.0.0140131 7772237

[171] Ulisse S Jannini EA Carosa E Piersanti D Graziano FM D'Armiento M. Inhibition of aromatase activity in rat Sertoli cells by thyroid hormone. J Endocrinol. (1994) 140:431–6. 10.1677/joe.0.1400431 8182371

[172] Holsberger DR Kiesewetter SE Cooke PS. Regulation of neonatal Sertoli cell development by thyroid hormone receptor alpha1. Biol Reprod. (2005) 73:396–403. 10.1095/biolreprod.105.041426 15858214

[173] Fumel B Guerquin MJ Livera G Staub C Magistrini M Gauthier C Thyroid hormone limits postnatal Sertoli cell proliferation *in vivo* by activation of its alpha1 isoform receptor (TRalpha1) present in these cells and by regulation of Cdk4/JunD/c-myc mRNA levels in mice. Biol Reprod. (2012) 87:16, 1–9. 10.1095/biolreprod.111.098418 22539677

[174] Fumel B Roy S Fouchecourt S Livera G Parent AS Casas F. Depletion of the p43 mitochondrial T3 receptor increases Sertoli cell proliferation in mice. PLoS ONE. (2013) 8:e74015. 10.1371/journal.pone.0074015 24040148 PMC3767600

[175] Gilleron J Nebout M Scarabelli L Senegas-Balas F Palmero S Segretain D. A potential novel mechanism involving connexin 43 gap junction for control of sertoli cell proliferation by thyroid hormones. J Cell Physiol. (2006) 209:153–61. 10.1002/jcp.20716 16823880

[176] Sridharan S Simon L Meling DD Cyr DG Gutstein DE Fishman GI. Proliferation of adult sertoli cells following conditional knockout of the Gap junctional protein GJA1 (connexin 43) in mice. Biol Reprod. (2007) 76:804–12. 10.1095/biolreprod.106.059212 17229929

[177] Beumer TL Kiyokawa H Roepers-Gajadien HL van den Bos LA Lock TM Gademan IS. Regulatory role of p27kip1 in the mouse and human testis. Endocrinology. (1999) 140:1834–40. 10.1210/endo.140.4.6638 10098522

[178] Holsberger DR Jirawatnotai S Kiyokawa H Cooke PS. Thyroid hormone regulates the cell cycle inhibitor p27Kip1 in postnatal murine Sertoli cells. Endocrinology. (2003) 144:3732–8. 10.1210/en.2003-0389 12933641

[179] Hershko A Ciechanover A. The ubiquitin system. Annu Rev Biochem. (1998) 67:425–79. 10.1146/annurev.biochem.67.1.425 9759494

[180] Bornstein G Bloom J Sitry-Shevah D Nakayama K Pagano M Hershko A. Role of the SCFSkp2 ubiquitin ligase in the degradation of p21Cip1 in S phase. J Biol Chem. (2003) 278:25752–7. 10.1074/jbc.M301774200 12730199

[181] Sun Y Yang W Luo H Wang X Chen Z Zhang J. Thyroid hormone inhibits the proliferation of piglet Sertoli cell via PI3K signaling pathway. Theriogenology. (2015) 83:86–94. 10.1016/j.theriogenology.2014.08.003 25284282

[182] Imamura K Ogura T Kishimoto A Kaminishi M Esumi H. Cell cycle regulation via p53 phosphorylation by a 5'-AMP activated protein kinase activator, 5-aminoimidazole- 4-carboxamide-1-beta-D-ribofuranoside, in a human hepatocellular carcinoma cell line. Biochem Biophys Res Commun. (2001) 287:562–7. 10.1006/bbrc.2001.5627 11554766

[183] Rattan R Giri S Singh AK Singh I. 5-Aminoimidazole-4-carboxamide-1-beta-D-ribofuranoside inhibits cancer cell proliferation *in vitro* and *in vivo* via AMP-activated protein kinase. J Biol Chem. (2005) 280:39582–93. 10.1074/jbc.M507443200 16176927

[184] Shaw RJ. LKB1 and AMP-activated protein kinase control of mTOR signalling and growth. Acta Physiol. (2009) 196:65–80. 10.1111/j.1748-1716.2009.01972.x 19245654 PMC2760308

[185] Yamauchi M Kambe F Cao X Lu X Kozaki Y Oiso Y. Thyroid hormone activates adenosine 5'-monophosphate-activated protein kinase via intracellular calcium mobilization and activation of calcium/calmodulin-dependent protein kinase kinase-beta. Mol Endocrinol. (2008) 22:893–903. 10.1210/me.2007-0249 18187603 PMC5419547

[186] Irrcher I Walkinshaw DR Sheehan TE Hood DA. Thyroid hormone (T3) rapidly activates p38 and AMPK in skeletal muscle *in vivo*. J Appl Physiol. (2008) 104:178–85. 10.1152/japplphysiol.00643.2007 17962579

[187] Videla LA Fernandez V Cornejo P Vargas R Morales P Ceballo J T(3)-induced liver AMP-activated protein kinase signaling: redox dependency and upregulation of downstream targets. World J Gastroenterol. (2014) 20:17416–25. 10.3748/wjg.v20.i46.17416 25516653 PMC4265600

[188] McBurney MW Yang X Jardine K Hixon M Boekelheide K Webb JR. The mammalian SIR2alpha protein has a role in embryogenesis and gametogenesis. Mol Cell Biol. (2003) 23:38–54. 10.1128/MCB.23.1.38-54.2003 12482959 PMC140671

[189] Gorga A Rindone GM Regueira M Pellizzari EH Camberos MC Cigorraga SB Effect of resveratrol on Sertoli cell proliferation. J Cell Biochem. (2018) 119:10131–42. 10.1002/jcb.27350 30129147

[190] Singh BK Sinha RA Zhou J Xie SY You SH Gauthier K. FoxO1 deacetylation regulates thyroid hormone-induced transcription of key hepatic gluconeogenic genes. J Biol Chem. (2013) 288:30365–72. 10.1074/jbc.M113.504845 23995837 PMC3798501

[191] Thakran S Sharma P Attia RR Hori RT Deng X Elam MB. Role of sirtuin 1 in the regulation of hepatic gene expression by thyroid hormone. J Biol Chem. (2013) 288:807–18. 10.1074/jbc.M112.437970 23209300 PMC3543030

[192] Vazquez-Anaya G Martinez B Sonanez-Organis JG Nakano D Nishiyama A Ortiz RM. Exogenous thyroxine improves glucose intolerance in insulin-resistant rats. J Endocrinol. (2017) 232:501–11. 10.1530/JOE-16-0428 27980001 PMC5419047

[193] Hess RA Carnes K The role of estrogen in testis and the male reproductive tract: a review and species comparison. Anim Reprod. (2004) 1:5–30.

[194] Carreau S Hess RA. Oestrogens and spermatogenesis. Philos Trans R Soc Lond B Biol Sci. (2010) 365:1517–35. 10.1098/rstb.2009.0235 20403867 PMC2871919

[195] Santen RJ Brodie H Simpson ER Siiteri PK Brodie A. History of aromatase: saga of an important biological mediator and therapeutic target. Endocr Rev. (2009) 30:343–75. 10.1210/er.2008-0016 19389994

[196] Free MJ Jaffe RA. Collection of rete testis fluid from rats without previous efferent duct ligation. Biol Reprod. (1979) 20:269–78. 10.1095/biolreprod20.2.269 454738

[197] Tsai-Morris CH Aquilano DR Dufau ML. Cellular localization of rat testicular aromatase activity during development. Endocrinology. (1985) 116:38–46. 10.1210/endo-116-1-38 2981072

[198] Nitta H Bunick D Hess RA Janulis L Newton SC Millette CF. Germ cells of the mouse testis express P450 aromatase. Endocrinology. (1993) 132:1396–401. 10.1210/endo.132.3.8440194 8440194

[199] Papadopoulos V Carreau S Szerman-Joly E Drosdowsky MA Dehennin L Scholler R. Rat testis 17 beta-estradiol: identification by gas chromatography-mass spectrometry and age related cellular distribution. J Steroid Biochem. (1986) 24:1211–6. 10.1016/0022-4731(86)90385-7 3736047

[200] Rappaport MS Smith EP. Insulin-like growth factor I inhibits aromatization induced by follice-stimulating hormone in rat sertoli cell culture. Biol Reprod. (1996) 54:446–52. 10.1095/biolreprod54.2.446 8788198

[201] Le Magueresse B Jegou B. *In vitro* effects of germ cells on the secretory activity of Sertoli cells recovered from rats of different ages. Endocrinology. (1988) 122:1672–80. 10.1210/endo-122-4-1672 2831038

[202] Levin ER Plasma membrane estrogen receptors. Trends Endocrinol Metab. (2009) 20:477–82. 10.1016/j.tem.2009.06.009 19783454 PMC3589572

[203] Wang Z Zhang X Shen P Loggie BW Chang Y Deuel TF. A variant of estrogen receptor-{alpha}, hER-{alpha}36: transduction of estrogen- and antiestrogen-dependent membrane-initiated mitogenic signaling. Proc Natl Acad Sci USA. (2006) 103:9063–8. 10.1073/pnas.0603339103 16754886 PMC1482566

[204] Li L Hisamoto K Kim KH Haynes MP Bauer PM Sanjay A. Variant estrogen receptor-c-Src molecular interdependence and c-Src structural requirements for endothelial NO synthase activation. Proc Natl Acad Sci USA. (2007) 104:16468–73. 10.1073/pnas.0704315104 17921256 PMC2034248

[205] Chimento A Sirianni R Casaburi I Pezzi V. GPER Signaling in Spermatogenesis and Testicular Tumors. Front Endocrinol. (2014) 5:30. 10.3389/fendo.2014.00030 24639669 PMC3944538

[206] Evinger AJ III Levin ER. Requirements for estrogen receptor alpha membrane localization and function. Steroids. (2005) 70:361–3. 10.1016/j.steroids.2005.02.015 15862818

[207] Fisher JS Millar MR Majdic G Saunders PT Fraser HM Sharpe RM. Immunolocalisation of oestrogen receptor-alpha within the testis and excurrent ducts of the rat and marmoset monkey from perinatal life to adulthood. J Endocrinol. (1997) 153:485–95. 10.1677/joe.0.1530485 9204003

[208] van Pelt AM de Rooij DG van der Burg B van der Saag PT Gustafsson JA Kuiper GG. Ontogeny of estrogen receptor-beta expression in rat testis. Endocrinology. (1999) 140:478–83. 10.1210/endo.140.1.6438 9886860

[209] Bois C Delalande C Nurmio M Parvinen M Zanatta L Toppari J. Age- and cell-related gene expression of aromatase and estrogen receptors in the rat testis. J Mol Endocrinol. (2010) 45:147–59. 10.1677/JME-10-0041 20554652

[210] Lucas TFG Lazari MFM Porto CS. Differential role of the estrogen receptors ESR1 and ESR2 on the regulation of proteins involved with proliferation and differentiation of Sertoli cells from 15-day-old rats. Mol Cell Endocrinol. (2014) 382:84–96. 10.1016/j.mce.2013.09.015 24056172

[211] Olde B Leeb-Lundberg LM. GPR30/GPER1: searching for a role in estrogen physiology. Trends Endocrinol Metab. (2009) 20:409–16. 10.1016/j.tem.2009.04.006 19734054

[212] Otto C Fuchs I Kauselmann G Kern H Zevnik B Andreasen P GPR30 does not mediate estrogenic responses in reproductive organs in mice. Biol Reprod. (2009) 80:34–41. 10.1095/biolreprod.108.071175 18799753

[213] Isensee J Meoli L Zazzu V Nabzdyk C Witt H Soewarto D. Expression pattern of G protein-coupled receptor 30 in LacZ reporter mice. Endocrinology. (2009) 150:1722–30. 10.1210/en.2008-1488 19095739

[214] Sirianni R Chimento A Ruggiero C De Luca A Lappano R Ando S. The novel estrogen receptor, G protein-coupled receptor 30, mediates the proliferative effects induced by 17beta-estradiol on mouse spermatogonial GC-1 cell line. Endocrinology. (2008) 149:5043–51. 10.1210/en.2007-1593 18566133

[215] Chimento A Sirianni R Delalande C Silandre D Bois C Ando S. 17 beta-estradiol activates rapid signaling pathways involved in rat pachytene spermatocytes apoptosis through GPR30 and ER alpha. Mol Cell Endocrinol. (2010) 320:136–44. 10.1016/j.mce.2010.01.035 20132863

[216] Lucas TF Royer C Siu ER Lazari MF Porto CS. Expression and signaling of G protein-coupled estrogen receptor 1 (GPER) in rat sertoli cells. Biol Reprod. (2010) 83:307–17. 10.1095/biolreprod.110.084160 20445128

[217] Lucas TF Pimenta MT Pisolato R Lazari MF Porto CS. 17beta-estradiol signaling and regulation of Sertoli cell function. Spermatogenesis. (2011) 1:318–24. 10.4161/spmg.1.4.18903 22332115 PMC3271643

[218] Atanassova N McKinnell C Walker M Turner KJ Fisher JS Morley M. Permanent effects of neonatal estrogen exposure in rats on reproductive hormone levels, Sertoli cell number, and the efficiency of spermatogenesis in adulthood. Endocrinology. (1999) 140:5364–73. 10.1210/endo.140.11.7108 10537168

[219] Berger T Kentfield L Roser JF Conley A. Stimulation of Sertoli cell proliferation: defining the response interval to an inhibitor of estrogen synthesis in the boar. Reproduction. (2012) 143:523–9. 10.1530/REP-11-0464 22367591

[220] Berger T Conley AJ Van Klompenberg M Roser JF Hovey RC. Increased testicular Sertoli cell population induced by an estrogen receptor antagonist. Mol Cell Endocrinol. (2013) 366:53–8. 10.1016/j.mce.2012.11.011 23178163

[221] Li X Nokkala E Yan W Streng T Saarinen N Warri A. Altered structure and function of reproductive organs in transgenic male mice overexpressing human aromatase. Endocrinology. (2001) 142:2435–42. 10.1210/endo.142.6.8211 11356692

[222] Robertson KM O'Donnell L Jones ME Meachem SJ Boon WC Fisher CR. Impairment of spermatogenesis in mice lacking a functional aromatase (cyp 19) gene. Proc Natl Acad Sci USA. (1999) 96:7986–91. 10.1073/pnas.96.14.7986 10393934 PMC22174

[223] Lubahn DB Moyer JS Golding TS Couse JF Korach KS Smithies O Alteration of reproductive function but not prenatal sexual development after insertional disruption of the mouse estrogen receptor gene. Proc Natl Acad Sci USA. (1993) 90:11162–6. 10.1073/pnas.90.23.11162 8248223 PMC47942

[224] Korach KS Couse JF Curtis SW Washburn TF Lindzey J Kimbro KS. Estrogen receptor gene disruption: molecular characterization and experimental and clinical phenotypes. Recent Prog Horm Res. (1996) 51:159–86; discussion 86–8. 8701078

[225] Gould ML Hurst PR Nicholson HD. The effects of oestrogen receptors alpha and beta on testicular cell number and steroidogenesis in mice. Reproduction. (2007) 134:271–9. 10.1530/REP-07-0025 17660237

[226] Antal MC Krust A Chambon P Mark M. Sterility and absence of histopathological defects in nonreproductive organs of a mouse ERbeta-null mutant. Proc Natl Acad Sci USA. (2008) 105:2433–8. 10.1073/pnas.0712029105 18268329 PMC2268154

[227] Yang WR Zhu FW Zhang JJ Wang Y Zhang JH Lu C. PI3K/Akt activated by GPR30 and Src regulates 17beta-estradiol-induced cultured immature boar sertoli cells proliferation. Reprod Sci. (2017) 24:57–66. 10.1177/1933719116649696 27222231

[228] Cavazzini D Galdieri M Ottonello S. Retinoic acid synthesis in the somatic cells of rat seminiferous tubules. Biochim Biophys Acta. (1996) 1313:139–45. 10.1016/0167-4889(96)00065-1 8781561

[229] Deltour L Haselbeck RJ Ang HL Duester G. Localization of class I and class IV alcohol dehydrogenases in mouse testis and epididymis: potential retinol dehydrogenases for endogenous retinoic acid synthesis. Biol Reprod. (1997) 56:102–9. 10.1095/biolreprod56.1.102 9002638

[230] Vernet N Dennefeld C Rochette-Egly C Oulad-Abdelghani M Chambon P Ghyselinck NB. Retinoic acid metabolism and signaling pathways in the adult and developing mouse testis. Endocrinology. (2006) 147:96–110. 10.1210/en.2005-0953 16210368

[231] Guo X Morris P Gudas L. Follicle-stimulating hormone and leukemia inhibitory factor regulate Sertoli cell retinol metabolism. Endocrinology. (2001) 142:1024–32. 10.1210/endo.142.3.7996 11181515

[232] Kurlandsky SB Gamble MV Ramakrishnan R Blaner WS. Plasma delivery of retinoic acid to tissues in the rat. J Biol Chem. (1995) 270:17850–7. 10.1074/jbc.270.30.17850 7629087

[233] Raverdeau M Gely-Pernot A Feret B Dennefeld C Benoit G Davidson I. Retinoic acid induces Sertoli cell paracrine signals for spermatogonia differentiation but cell autonomously drives spermatocyte meiosis. Proc Natl Acad Sci USA. (2012) 109:16582–7. 10.1073/pnas.1214936109 23012458 PMC3478620

[234] Mitranond V Sobhon P Tosukhowong P Chindaduangrat W Cytological changes in the testes of vitamin-A-deficient rats. I. Quantitation of germinal cells in the seminiferous tubules. Acta Anatom. (1979) 103:159–68. 10.1159/000145007 419926

[235] Sobhon P Mitranond V Tosukhowong P Chindaduangrat W Cytological changes in the testes of vitamin-A-deficient rats. II. Ultrastructural study of the seminiferous tubules. Acta Anatom. (1979) 103:169–83. 10.1159/000145008 419927

[236] van Pelt AM de Rooij DG. Synchronization of the seminiferous epithelium after vitamin A replacement in vitamin A-deficient mice. Biol Reprod. (1990) 43:363–67. 10.1095/biolreprod43.3.363 2271719

[237] Hogarth CA Griswold MD. The key role of vitamin A in spermatogenesis. J Clin Invest. (2010) 120:956–62. 10.1172/JCI41303 20364093 PMC2846058

[238] Akmal KM Dufour JM Kim KH. Retinoic acid receptor alpha gene expression in the rat testis: potential role during the prophase of meiosis and in the transition from round to elongating spermatids. Biol Reprod. (1997) 56:549–56. 10.1095/biolreprod56.2.549 9116160

[239] Dufour JM Kim KH. Cellular and subcellular localization of six retinoid receptors in rat testis during postnatal development: identification of potential heterodimeric receptors. Biol Reprod. (1999) 61:1300–8. 10.1095/biolreprod61.5.1300 10529278

[240] Boulogne B Levacher C Durand P Habert R. Retinoic acid receptors and retinoid X receptors in the rat testis during fetal and postnatal development: immunolocalization and implication in the control of the number of gonocytes. Biol Reprod. (1999) 61:1548–57. 10.1095/biolreprod61.6.1548 10570002

[241] Nicholls PK Harrison CA Rainczuk KE Wayne Vogl A Stanton PG. Retinoic acid promotes Sertoli cell differentiation and antagonises activin-induced proliferation. Mol Cell Endocrinol. (2013) 377:33–43. 10.1016/j.mce.2013.06.034 23831638

[242] Lufkin T Lohnes D Mark M Dierich A Gorry P Gaub MP. High postnatal lethality and testis degeneration in retinoic acid receptor alpha mutant mice. Proc Natl Acad Sci USA. (1993) 90:7225–9. 10.1073/pnas.90.15.7225 8394014 PMC47109

[243] Lohnes D Kastner P Dierich A Mark M LeMeur M Chambon P. Function of retinoic acid receptor gamma in the mouse. Cell. (1993) 73:643–58. 10.1016/0092-8674(93)90246-M 8388780

[244] Gely-Pernot A Raverdeau M Celebi C Dennefeld C Feret B Klopfenstein M. Spermatogonia differentiation requires retinoic acid receptor gamma. Endocrinology. (2012) 153:438–49. 10.1210/en.2011-1102 22045663

[245] Kastner P Mark M Leid M Gansmuller A Chin W Grondona JM. Abnormal spermatogenesis in RXR beta mutant mice. Genes Dev. (1996) 10:80–92. 10.1101/gad.10.1.80 8557197

[246] Luo J Pasceri P Conlon RA Rossant J Giguere V. Mice lacking all isoforms of retinoic acid receptor beta develop normally and are susceptible to the teratogenic effects of retinoic acid. Mech Dev. (1995) 53:61–71. 10.1016/0925-4773(95)00424-6 8555112

[247] Krezel W Dupe V Mark M Dierich A Kastner P Chambon P. RXR gamma null mice are apparently normal and compound RXR alpha +/-/RXR beta -/-/RXR gamma -/- mutant mice are viable. Proc Natl Acad Sci USA. (1996) 93:9010–4. 10.1073/pnas.93.17.9010 8799145 PMC38586

[248] Kastner P Grondona JM Mark M Gansmuller A LeMeur M Decimo D. Genetic analysis of RXR alpha developmental function: convergence of RXR and RAR signaling pathways in heart and eye morphogenesis. Cell. (1994) 78:987–1003. 10.1016/0092-8674(94)90274-7 7923367

[249] Vernet N Dennefeld C Guillou F Chambon P Ghyselinck NB Mark M Prepubertal testis development relies on retinoic acid but not rexinoid receptors in Sertoli cells. EMBO J. (2006) 25:5816–25. 10.1038/sj.emboj.7601447 17124491 PMC1698894

[250] Hasegawa K Saga Y. Retinoic acid signaling in Sertoli cells regulates organization of the blood-testis barrier through cyclical changes in gene expression. Development. (2012) 139:4347–55. 10.1242/dev.080119 23095883

[251] Huang HF Yang CS Meyenhofer M Gould S Boccabella AV. Disruption of sustentacular (Sertoli) cell tight junctions and regression of spermatogenesis in vitamin-A-deficient rats. Acta Anatom. (1988) 133:10–5. 10.1159/000146606 3213399

[252] Morales A Cavicchia JC. Spermatogenesis and blood-testis barrier in rats after long-term Vitamin A deprivation. Tissue Cell. (2002) 34:349–55. 10.1016/S0040816602000356 12270261

[253] Chung SS Choi C Wang X Hallock L Wolgemuth DJ. Aberrant distribution of junctional complex components in retinoic acid receptor alpha-deficient mice. Microscopy Res Tech. (2010) 73:583–96. 10.1002/jemt.20797 19937743 PMC2877760

[254] Tsong SD Phillips D Halmi N Liotta AS Margioris A Bardin CW. ACTH and beta-endorphin-related peptides are present in multiple sites in the reproductive tract of the male rat. Endocrinology. (1982) 110:2204–6. 10.1210/endo-110-6-2204 6280990

[255] Bardin CW Shaha C Mather J Salomon Y Margioris AN Liotta AS. Identification and possible function of pro-opiomelanocortin-derived peptides in the testis. Ann N Y Acad Sci. (1984) 438:346–64. 10.1111/j.1749-6632.1984.tb38296.x 6100019

[256] Fabbri A Knox G Buczko E Dufau ML. Beta-endorphin production by the fetal Leydig cell: regulation and implications for paracrine control of Sertoli cell function. Endocrinology. (1988) 122:749–55. 10.1210/endo-122-2-749 2962854

[257] Fabbri A Tsai-Morris CH Luna S Fraioli F Dufau ML. Opiate receptors are present in the rat testis. Identification and localization in Sertoli cells. Endocrinology. (1985) 117:2544–6. 10.1210/endo-117-6-2544 2998740

[258] Jenab S Morris PL. Interleukin-6 regulation of kappa opioid receptor gene expression in primary sertoli cells. Endocrine. (2000) 13:11–5. 10.1385/ENDO:13:1:11 11051042

[259] Orth JM. FSH-induced Sertoli cell proliferation in the developing rat is modified by beta-endorphin produced in the testis. Endocrinology. (1986) 119:1876–8. 10.1210/endo-119-4-1876 2944740

[260] Orth JM Boehm R. Endorphin suppresses FSH-stimulated proliferation of isolated neonatal Sertoli cells by a pertussis toxin-sensitive mechanism. Anat Rec. (1990) 226:320–7. 10.1002/ar.1092260308 2139307

[261] da Silva VA Jr Vieira AC Pinto CF de Paula TA Palma MB Lins Amorim MJ. Neonatal treatment with naloxone increases the population of Sertoli cells and sperm production in adult rats. Reprod Nutr Dev. (2006) 46:157–66. 10.1051/rnd:2006001 16597421

[262] Fairley KF Barrie JU Johnson W. Sterility and testicular atrophy related to cyclophosphamide therapy. Lancet. (1972) 1:568–9. 10.1016/S0140-6736(72)90358-3 4110052

[263] Qureshi MS Pennington JH Goldsmith HJ Cox PE. Cyclophosphamide therapy and sterility. Lancet. (1972) 2:1290–1. 10.1016/S0140-6736(72)92657-8 4117814

[264] Meistrich ML. Effects of chemotherapy and radiotherapy on spermatogenesis in humans. Fertil Steril. (2013) 100:1180–6. 10.1016/j.fertnstert.2013.08.010 24012199 PMC3826884

[265] Rivkees SA Crawford JD. The relationship of gonadal activity and chemotherapy-induced gonadal damage. JAMA. (1988) 259:2123–5. 10.1001/jama.1988.03720140043031 3162285

[266] Nurmio M Keros V Lahteenmaki P Salmi T Kallajoki M Jahnukainen K. Effect of childhood acute lymphoblastic leukemia therapy on spermatogonia populations and future fertility. J Clin Endocrinol Metab. (2009) 94:2119–22. 10.1210/jc.2009-0060 19318447

[267] Wasilewski-Masker K Seidel KD Leisenring W Mertens AC Shnorhavorian M Ritenour CW. Male infertility in long-term survivors of pediatric cancer: a report from the childhood cancer survivor study. J Cancer Survivorship. (2014) 8:437–47. 10.1007/s11764-014-0354-6 24711092 PMC4276596

[268] Chow EJ Stratton KL Leisenring WM Oeffinger KC Sklar CA Donaldson SS. Pregnancy after chemotherapy in male and female survivors of childhood cancer treated between 1970 and 1999: a report from the Childhood Cancer Survivor Study cohort. Lancet Oncol. (2016) 17:567–76. 10.1016/S1470-2045(16)00086-3 27020005 PMC4907859

[269] Hou M Chrysis D Nurmio M Parvinen M Eksborg S Soder O. Doxorubicin induces apoptosis in germ line stem cells in the immature rat testis and amifostine cannot protect against this cytotoxicity. Cancer Res. (2005) 65:9999–10005. 10.1158/0008-5472.CAN-05-2004 16267025

[270] Tremblay AR Delbes G. *In vitro* study of doxorubicin-induced oxidative stress in spermatogonia and immature Sertoli cells. Toxicol Appl Pharmacol. (2018) 348:32–42. 10.1016/j.taap.2018.04.014 29660436

[271] Nurmio M Toppari J Kallio J Hou M Soder O Jahnukainen K. Functional *in vitro* model to examine cancer therapy cytotoxicity in maturing rat testis. Reprod Toxicol. (2009) 27:28–34. 10.1016/j.reprotox.2008.10.004 19027063

[272] Liu F Li XL Lin T He DW Wei GH Liu JH. The cyclophosphamide metabolite, acrolein, induces cytoskeletal changes and oxidative stress in Sertoli cells. Mol Biol Rep. (2012) 39:493–500. 10.1007/s11033-011-0763-9 21553225

[273] Smart E Lopes F Rice S Nagy B Anderson RA Mitchell RT. Chemotherapy drugs cyclophosphamide, cisplatin and doxorubicin induce germ cell loss in an in vitro model of the prepubertal testis. Sci Rep. (2018) 8:1773. 10.1038/s41598-018-19761-9 29379115 PMC5788858

[274] Faqi AS Klug A Merker HJ Chahoud I. Ganciclovir induces reproductive hazards in male rats after short-term exposure. Hum Exp Toxicol. (1997) 16:505–11. 10.1177/096032719701600905 9306137

[275] Narayana K. A purine nucleoside analogue-acyclovir [9-(2-hydroxyethoxymethyl)-9h-guanine] reversibly impairs testicular functions in mouse. J Toxicol Sci. (2008) 33:61–70. 10.2131/jts.33.61 18303185

[276] Nihi F Moreira D Santos Lourenco AC Gomes C Araujo SL Zaia RM. Testicular effects following *in utero* exposure to the antivirals acyclovir and ganciclovir in rats. Toxicol Sci. (2014) 139:220–33. 10.1093/toxsci/kfu024 24496639

[277] Qiu R Horvath A Stahlmann R. Effects of four nucleoside analogues used as antiviral agents on rat Sertoli cells (SerW3) *in vitro*. Arch Toxicol. (2016) 90:1975–81. 10.1007/s00204-016-1743-6 27224990

[278] Ben Maamar M Lesne L Hennig K Desdoits-Lethimonier C Kilcoyne KR Coiffec I. Ibuprofen results in alterations of human fetal testis development. Sci Rep. (2017) 7:44184. 10.1038/srep44184 28281692 PMC5345102

[279] Rossitto M Marchive C Pruvost A Sellem E Ghettas A Badiou S. Intergenerational effects on mouse sperm quality after in utero exposure to acetaminophen and ibuprofen. FASEB J. (2019) 33:339–57. 10.1096/fj.201800488RRR 29979629

[280] Romero R Erez O Huttemann M Maymon E Panaitescu B Conde-Agudelo A. Metformin, the aspirin of the 21st century: its role in gestational diabetes mellitus, prevention of preeclampsia and cancer, and the promotion of longevity. Am J Obstetr Gynecol. (2017) 217:282–302. 10.1016/j.ajog.2017.06.003 28619690 PMC6084482

[281] Moghetti P Castello R Negri C Tosi F Perrone F Caputo M. Metformin effects on clinical features, endocrine and metabolic profiles, and insulin sensitivity in polycystic ovary syndrome: a randomized, double-blind, placebo-controlled 6-month trial, followed by open, long-term clinical evaluation. J Clin Endocrinol Metab. (2000) 85:139–46. 10.1210/jcem.85.1.6293 10634377

[282] Vanky E Zahlsen K Spigset O Carlsen SM. Placental passage of metformin in women with polycystic ovary syndrome. Fertil Steril. (2005) 83:1575–8. 10.1016/j.fertnstert.2004.11.051 15866611

[283] Brufani C Crino A Fintini D Patera PI Cappa M Manco M. Systematic review of metformin use in obese nondiabetic children and adolescents. Horm Res Paediatr. (2013) 80:78–85. 10.1159/000353760 23899569

[284] Adeyemo MA McDuffie JR Kozlosky M Krakoff J Calis KA Brady SM. Effects of metformin on energy intake and satiety in obese children. Diabetes Obes Metab. (2015) 17:363–70. 10.1111/dom.12426 25483291 PMC4357555

[285] Smith JD Mills E Carlisle SE. Treatment of Pediatric Type 2 Diabetes. Ann Pharmacother. (2016) 50:768–77. 10.1177/1060028016655179 27307414

[286] Isakovic A Harhaji L Stevanovic D Markovic Z Sumarac-Dumanovic M Starcevic V. Dual antiglioma action of metformin: cell cycle arrest and mitochondria-dependent apoptosis. Cell Mol Life Sci. (2007) 64:1290–302. 10.1007/s00018-007-7080-4 17447005 PMC11136022

[287] Ben Sahra I Laurent K Loubat A Giorgetti-Peraldi S Colosetti P Auberger P. The antidiabetic drug metformin exerts an antitumoral effect *in vitro* and *in vivo* through a decrease of cyclin D1 level. Oncogene. (2008) 27:3576–86. 10.1038/sj.onc.1211024 18212742

[288] Rattan R Giri S Hartmann LC Shridhar V. Metformin attenuates ovarian cancer cell growth in an AMP-kinase dispensable manner. J Cell Mol Med. (2011) 15:166–78. 10.1111/j.1582-4934.2009.00954.x 19874425 PMC3822503

[289] Tartarin P Moison D Guibert E Dupont J Habert R Rouiller-Fabre V. Metformin exposure affects human and mouse fetal testicular cells. Hum Reprod. (2012) 27:3304–14. 10.1093/humrep/des264 22811314

[290] Faure M Guibert E Alves S Pain B Rame C Dupont J. The insulin sensitiser metformin regulates chicken Sertoli and germ cell populations. Reproduction. (2016) 151:527–38. 10.1530/REP-15-0565 26917452

[291] Rindone GM Gorga A Regueira M Pellizzari EH Cigorraga SB Galardo MN. Metformin counteracts the effects of FSH on rat Sertoli cell proliferation. Reproduction. (2018) 156:93–101. 10.1530/REP-18-0233 29789441

[292] Madiraju AK Erion DM Rahimi Y Zhang XM Braddock DT Albright RA. Metformin suppresses gluconeogenesis by inhibiting mitochondrial glycerophosphate dehydrogenase. Nature. (2014) 510:542–6. 10.1038/nature13270 24847880 PMC4074244

[293] Owen MR Doran E Halestrap AP. Evidence that metformin exerts its anti-diabetic effects through inhibition of complex 1 of the mitochondrial respiratory chain. Biochem J. (2000) 348 (Pt 3):607–14. 10.1042/bj3480607 10839993 PMC1221104

[294] Viollet B Guigas B Sanz Garcia N Leclerc J Foretz M Andreelli F. Cellular and molecular mechanisms of metformin: an overview. Clin Sci. (2012) 122:253–70. 10.1042/CS20110386 22117616 PMC3398862

[295] Garcia MS Constantino DH Silva AP Perobelli JE. Fish pollutants MeHg and Aroclor cause permanent structural damage in male gonads and kidneys after prepubertal exposure. Int J Exp Pathol. (2016) 97:360–8. 10.1111/iep.12200 27917541 PMC5206816

[296] Mylchreest E Cattley RC Foster PM. Male reproductive tract malformations in rats following gestational and lactational exposure to Di(n-butyl) phthalate: an antiandrogenic mechanism? Toxicol Sci. (1998) 43:47–60. 10.1093/toxsci/43.1.47 9629619

[297] Wisniewski P Romano RM Kizys MM Oliveira KC Kasamatsu T Giannocco G. Adult exposure to bisphenol A (BPA) in Wistar rats reduces sperm quality with disruption of the hypothalamic-pituitary-testicular axis. Toxicology. (2015) 329:1–9. 10.1016/j.tox.2015.01.002 25575453

[298] Wilker C Johnson L Safe S. Effects of developmental exposure to indole-3-carbinol or 2,3,7,8-tetrachlorodibenzo-p-dioxin on reproductive potential of male rat offspring. Toxicol. Appl Pharmacol. (1996) 141:68–75. 10.1016/S0041-008X(96)80010-X 8917677

[299] Axelstad M Hass U Scholze M Christiansen S Kortenkamp A Boberg J. EDC IMPACT: reduced sperm counts in rats exposed to human relevant mixtures of endocrine disrupters. Endocr Connect. (2018) 7:139–48. 10.1530/EC-17-0307 29203468 PMC5776667

[300] Sekaran S Balaganapathy P Parsanathan R Elangovan S Gunashekar J Bhat FA. Lactational exposure of phthalate causes long-term disruption in testicular architecture by altering tight junctional and apoptotic protein expression in Sertoli cells of first filial generation pubertal Wistar rats. Hum Exp Toxicol. (2015) 34:575–90. 10.1177/0960327114555926 25352649

[301] Sedha S Kumar S Shukla S. Role of oxidative stress in male reproductive dysfunctions with reference to phthalate compounds. Urol J. (2015) 12:2304–16. 10.22037/uj.v12i5.3009 26571312

[302] Cheng CY Wong EW Lie PP Li MW Su L Siu ER. Environmental toxicants and male reproductive function. Spermatogenesis. (2011) 1:2–13. 10.4161/spmg.1.1.13971 21866273 PMC3158642

[303] Yao PL Lin YC Sawhney P Richburg JH. Transcriptional regulation of FasL expression and participation of sTNF-alpha in response to sertoli cell injury. J Biol Chem. (2007) 282:5420–31. 10.1074/jbc.M609068200 17192273

[304] Wang M Su P. The role of the Fas/FasL signaling pathway in environmental toxicant-induced testicular cell apoptosis: an update. Syst Biol Reprod Med. (2018) 64:93–102. 10.1080/19396368.2017.1422046 29299971

[305] Li LH Jester WF Jr Orth JM. Effects of relatively low levels of mono-(2-ethylhexyl) phthalate on cocultured Sertoli cells and gonocytes from neonatal rats. Toxicol Appl Pharmacol. (1998) 153:258–65. 10.1006/taap.1998.8550 9878596

[306] Li LH Jester WF Jr Laslett AL Orth JM. A single dose of Di-(2-ethylhexyl) phthalate in neonatal rats alters gonocytes, reduces sertoli cell proliferation, and decreases cyclin D2 expression. Toxicol Appl Pharmacol. (2000) 166:222–9. 10.1006/taap.2000.8972 10906286

[307] Zhang L Gao M Zhang T Chong T Wang Z Zhai X. Protective effects of genistein against Mono-(2-ethylhexyl) phthalate-induced oxidative damage in prepubertal sertoli cells. Biomed Res Int. (2017) 2017:2032697. 10.1155/2017/2032697 29259978 PMC5702931

[308] Yin X Ma T Han R Ding J Zhang H Han X. MiR-301b-3p/3584–5p enhances low-dose mono-n-butyl phthalate (MBP)-induced proliferation by targeting Rasd1 in Sertoli cells. Toxicol In Vitro. (2018) 47:79–88. 10.1016/j.tiv.2017.11.009 29162477

[309] Salian S Doshi T Vanage G. Perinatal exposure of rats to bisphenol A affects fertility of male offspring–an overview. Reprod Toxicol. (2011) 31:359–62. 10.1016/j.reprotox.2010.10.008 20965246

[310] Ge LC Chen ZJ Liu H Zhang KS Su Q Ma XY. Signaling related with biphasic effects of bisphenol A (BPA) on Sertoli cell proliferation: a comparative proteomic analysis. Biochim Biophys Acta. (2014) 1840:2663–73. 10.1016/j.bbagen.2014.05.018 24909818

[311] Johnson L Staub C Silge RL Harris MW Chapin RE. The pesticide methoxychlor given orally during the perinatal/juvenile period, reduced the spermatogenic potential of males as adults by reducing their Sertoli cell number. Reprod Nutr Dev. (2002) 42:573–80. 10.1051/rnd:2002043 12625421

[312] Wang BJ Zheng WL Feng NN Wang T Zou H Gu JH. The effects of autophagy and PI3K/AKT/m-TOR signaling pathway on the cell-cycle arrest of rats primary sertoli cells induced by zearalenone. Toxins. (2018) 10:398. 10.3390/toxins10100398 30274213 PMC6215106

[313] Ness DK Schantz SL Moshtaghian J Hansen LG. Effects of perinatal exposure to specific PCB congeners on thyroid hormone concentrations and thyroid histology in the rat. Toxicol Lett. (1993) 68:311–23. 10.1016/0378-4274(93)90023-Q 8516785

[314] Cooke PS Zhao YD Hansen LG. Neonatal polychlorinated biphenyl treatment increases adult testis size and sperm production in the rat. Toxicol Appl Pharmacol. (1996) 136:112–7. 10.1006/taap.1996.0013 8560463

[315] Kim IS. Effects of exposure of lactating female rats to polychlorinated biphenyls (Pcbs) on testis weight, sperm production and sertoli cell numbers in the adult male offspring. J Vet Med Sci. (2001) 63:5–9. 10.1292/jvms.63.5 11217063

[316] Fiorini C Tilloy-Ellul A Chevalier S Charuel C Pointis G. Sertoli cell junctional proteins as early targets for different classes of reproductive toxicants. Reprod Toxicol. (2004) 18:413–21. 10.1016/j.reprotox.2004.01.002 15082077

[317] Aravindakshan J Cyr DG. Nonylphenol alters connexin 43 levels and connexin 43 phosphorylation via an inhibition of the p38-mitogen-activated protein kinase pathway. Biol Reprod. (2005) 72:1232–40. 10.1095/biolreprod.104.038596 15647452

[318] Pointis G Gilleron J Carette D Segretain D. Testicular connexin 43, a precocious molecular target for the effect of environmental toxicants on male fertility. Spermatogenesis. (2011) 1:303–17. 10.4161/spmg.1.4.18392 22332114 PMC3271642

